# *Helicobacter pylori* employs a general protein glycosylation system for the modification of outer membrane adhesins

**DOI:** 10.1080/19490976.2022.2130650

**Published:** 2022-10-07

**Authors:** Kai-Wen Teng, Kai-Siang Hsieh, Ji-Shiuan Hung, Chun-Jen Wang, En-Chi Liao, Pei-Chun Chen, Ying-Hsuan Lin, Deng-Chyang Wu, Chun-Hung Lin, Wen-Ching Wang, Hong-Lin Chan, Shau-Ku Huang, Mou-Chieh Kao

**Affiliations:** a Institute of Molecular Medicine, College of Life Science, National Tsing Hua University, Hsinchu, Taiwan; b Institute of Bioinformatics and Structural Biology, College of Life Science, National Tsing Hua University, Hsinchu, Taiwan; c Department of Medical Science, College of Life Science, National Tsing Hua University, Hsinchu, Taiwan; d Division of Gastroenterology, Department of Internal Medicine, Kaohsiung Medical University Hospital, Kaohsiung Medical University, Kaohsiung, Taiwan; e Institute of Biological Chemistry, Academia Sinica, Taipei, Taiwan; f Institute of Molecular and Cellular Biology, College of Life Science, National Tsing Hua University, Hsinchu, Taiwan; g Department of Life Science, College of Life Science, National Tsing Hua University, Hsinchu, Taiwan; h National Institute of Environmental Health Sciences, National Health Research Institutes, Zhunan, Taiwan

**Keywords:** Helicobacter pylori, lipopolysaccharide, protein glycosylation, outer membrane adhesin, BabA, BabB, AlpA, AlpB, bacterial virulence, drug target

## Abstract

*Helicobacter pylori* infection is associated with the development of several gastric diseases including gastric cancer. To reach a long-term colonization in the host stomach, *H. pylori* employs multiple outer membrane adhesins for binding to the gastric mucosa. However, due to the redundancy of adhesins that complement the adhesive function of bacteria, targeting each individual adhesin alone usually achieves nonideal outcomes for preventing bacterial adhesion. Here, we report that key adhesins AlpA/B and BabA/B in *H. pylori* are modified by glycans and display a two-step molecular weight upshift pattern from the cytoplasm to the inner membrane and from the inner membrane to the outer membrane. Nevertheless, this upshift pattern is missing when the expression of some enzymes related to lipopolysaccharide (LPS) biosynthesis, including the LPS O-antigen assembly and ligation enzymes WecA, Wzk, and WaaL, is disrupted, indicating that the underlying mechanisms and the involved enzymes for the adhesin glycosylation are partially shared with the LPS biosynthesis. Loss of the adhesin glycosylation not only reduces the protease resistance and the stability of the tested adhesins but also changes the adhesin-binding ability. In addition, mutations in the LPS biosynthesis cause a significant reduction in bacterial adhesion in the *in vitro* cell-line model. The current findings reveal *that H. pylori* employs a general protein glycosylation system related to LPS biosynthesis for adhesin modification and its biological significance. The enzymes required for adhesin glycosylation rather than the adhesins themselves are potentially better drug targets for preventing or treating *H. pylori* infection.

## Introduction

*Helicobacter pylori* (*H. pylori*), a gram-negative bacterium, and also an etiological agent for many gastrointestinal diseases infects about half of the global population.^1^ It is a major gastric pathogen and possesses multiple virulence factors responsible for environmental adaptation and colonization, which cause infected humans to develop several gastric-related diseases, such as gastritis, peptic ulcer, gastric cancer, and mucosa-associated lymphoid tissue (MALT) lymphoma.^1–3^

Proteins belonging to the *Helicobacter* outer membrane protein (HOP) family are proposed to play a pivotal role in bacterial adhesion and successful colonization.^4^ Some of these outer membrane proteins, including the adhesins BabA, BabB, AlpA, AlpB, and SabA, have been extensively studied. For example, blood group antigen-binding adhesins A and B (BabA/B) are porin-like proteins (79.1 and 77.7 kDa, respectively, according to the prediction). BabA specifically binds to the blood group O antigen Lewis B and its related H1 antigen, which are mostly found in gastric epithelial cells and mucins.^5–8^ Recently, the 3D structure of the BabA protein bound to Lewis B antigen was resolved by X-ray crystallography, and BabA contains a beta-strand motif in the N-terminal domain and forms a crown-shaped hollow structure that interacts with the Lewis B antigen through hydrogen bonding.^9,10^ Adherence-associated lipoproteins A and B (AlpA/B, 55.9 and 57 kDa, respectively, according to the prediction) are suggested to be involved in the adhesion process. AlpA and AlpB share a high degree of sequence homology, and both of their coding sequences are located in an operon, suggesting that they should be controlled and transcribed together.^11^ The binding specificity of AlpA to laminin has been identified, and studies have shown that AlpA/B disruption mutants fail to colonize the surface of human, gerbil, and Guinea pig epithelial cells, strongly suggesting their contribution to *H. pylori* adhesion.^12–15^

Since adhesion is a critical step in the process of bacterial infection, blocking the adherent function of adhesins is a feasible strategy to prevent *H. pylori* colonization. However, the adherent ability of *H. pylori* is only partially reduced when a single adhesin is disrupted or the binding ability of an adhesin is inhibited by chemical drugs.^10,14^ A possible interpretation of these nonideal results may be the complex adhesive interaction of *H. pylori* with its host cells and the redundancy of adhesins with compensatory adhesive functions in this bacterium. That is, the deficiency of an adhesin is compensated by other adhesins, and the adherence of *H. pylori* is complemented to a competent level for subsequent infection.

Recently, we found that the disruption of genes associated with lipopolysaccharide (LPS) biosynthesis in *H. pylori* not only impaired LPS formation and bacterial virulence but also surprisingly caused a substantial reduction in bacterial adhesion.^16–18^ These findings indicate that, in addition to functioning directly as an adherent molecule through the LPS structure itself, the proteins involved in LPS biosynthesis may be linked to the bacterial adhesion process through an unknown mechanism. Furthermore, because outer membrane adhesins rather than LPS are generally considered the major adhesion molecules involved in *H. pylori* infection,^19^ this finding aroused our interest in exploring the connections between LPS biosynthesis, adhesins, and subsequent bacterial adhesion.

Here, our results show that the key adhesins AlpA, AlpB, BabA, and BabB are modified by glycans. The disruption of several genes involved in glycan biosynthesis and translocation of LPS O-antigen alter the electrophoretic mobility of the tested adhesins, suggesting that enzymes encoded by these genes are not only involved in LPS biosynthesis but also shared with a general protein glycosylation system identified in the present study. Disruption of adhesin glycosylation leads to significant reductions in adhesin resistance to proteases, protein stability, and adhesin binding ability. In this study, protein glycosylation was confirmed to be crucial for adhesin function. Since the disruption of protein glycosylation, as shown here for key adhesins, induces comprehensive defects in *H. pylori*, the enzymes involved in the protein glycosylation pathway may be suitable drug targets for preventing or treating *H. pylori* infection.

## Results

### Key adhesins in *H.* *pylori* are modified by glycans

**Figure 1.**
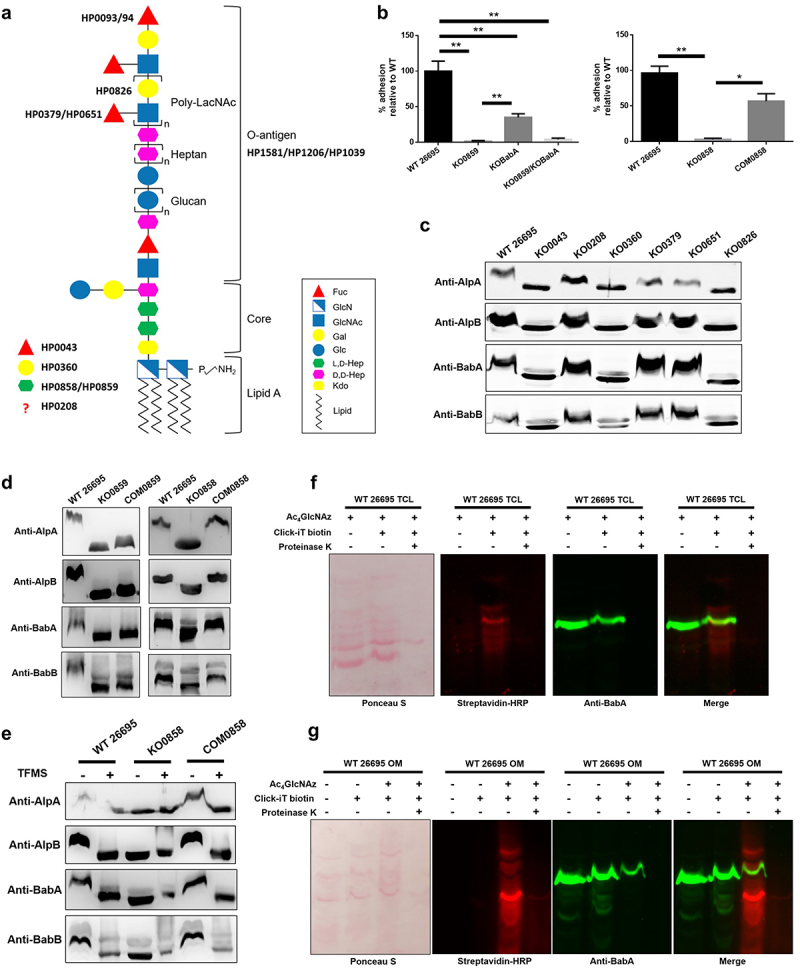
The glycosylation of key adhesins in *H. pylori* is associated with LPS biosynthesis. (a) The redefined LPS structure of *H. pylori* wild-type strains 26695 and G27 and the corresponding enzymes for monosaccharide biosynthesis and LPS biosynthesis being tested in this study. The O-antigen synthesis is initiated by a glycosyltransferase (HP1581) on the undecaprenyl phosphate (UndP) lipid carrier in the cytoplasm. After successive actions of a series of other glycosyltransferases, the resulting UndP-linked O-antigen is translocated by a flippase (HP1206) to the periplasm, where the translocated O-antigen is then transferred to the lipid A-core by an O-antigen ligase (HP1039). (b) The relative percentage of adherent bacteria after an incubation with AGS cells. The *H. pylori* wild-type strain 26695, *HP0859* knockout mutant, *babA* knockout mutant, *HP0859*/*babA* double knockout mutant, *HP0858* knockout mutant and *HP0858* knockout complementary mutant were co-incubated with AGS cells at an MOI of 100. The adhesion of various knockout mutants was statistically compared to that of the wild-type strain 26695 (unpaired, two-tailed Student’s t test; n = 3; *p < .05 and **p < .01). (c-e) Electrophoretic migration of the tested adhesins. The tested adhesins were separated on 12% SDS–PAGE gels for AlpA and AlpB or 10% SDS–PAGE gels for BabA and BabB. (c and d) Outer membrane protein samples from different *H. pylori* strains were extracted and analyzed using immunoblotting with AlpA-, AlpB-, BabA- and BabB-specific antibodies. (e) Outer membrane protein samples from the wild-type strain 26695, *HP0858* knockout mutant and *HP0858* knockout complementary mutant were deglycosylated with TFMS and subjected to immunoblotting with AlpA-, AlpB-, BabA- and BabB-specific antibodies. The *HP0858* knockout mutant was used as the negative control. (f and g) Metabolic labeling of proteins from *H. pylori* with an azide-containing sugar Ac_4_GlcNAz. Total cell lysates (f) or outer membrane samples (g) from regular- or Ac4GlcNAz-cultivated *H. pylori* wild-type strain 26695 were treated with or without an alkyne-biotin probe (Click-iT biotin), followed by digestion with or without 5 μg/mL proteinase K for 30 minutes. The resulting samples were subjected to immunoblotting with streptavidin-HRP and BabA-specific antibodies, respectively. WT, wild-type strain; KO, knockout mutant; COM, knockout complementary mutant; TCL, total cell lysate; OM, outer membrane.

In the beginning of this study, several mutants with disruption in the genes associated with LPS biosynthesis in different regions of LPS were generated, and the successful construction of these mutants was further confirmed by silver staining of the extracted LPS samples (Figure 1a, Supplementary Table S1, and Supplementary Figure S1). Next, to explore the association between LPS biosynthesis and adherence in *H. pylori*, we evaluated and compared the functional importance of the LPS core biosynthetic enzyme HP0859 and the key outer membrane adhesin BabA in bacterial adherence. The *HP0859* (codes for an epimerase HldD that can convert ADP-D-*glycero*-D-*manno* heptose to ADP-L-*glycero*-D-*manno* heptose; Supplementary Table S1) and *babA* knockout mutants were constructed and subjected to adhesion analysis, and both HP0859 and BabA deficiency exerted substantial effects on bacterial adherence. However, the degree of adhesion loss resulting from *HP0859* disruption was much more remarkable than that caused by *babA* knockout mutation (Figure 1b). Interestingly, a significant reduction in bacterial adhesion was also observed for the *HP0858* (codes for a bifunctional protein that can catalyze the phosphorylation of D,D-heptose-7-phosphate and the ADP transfer to D,D-heptose-1-phosphate; Supplementary Table S1) knockout mutant, in which its complementary mutant was also included in the test to exclude the possibility of a polar effect (Figure 1b). This result not only confirms that LPS biosynthesis is indeed associated with the adhesive ability of *H. pylori* but also implies that its contribution to *H. pylori* adhesion is even more significant than that of BabA through either a direct or an indirect mechanism. To further examine the possible relationship between LPS biosynthesis and adhesins, adhesins AlpA, AlpB, BabA, and BabB from the wild-type strain 26695 and LPS-disrupted mutants were detected using immunoblotting. SabA was not chosen because its expression is controlled by phase variation and inactivated (out-of-frame allele) in the tested strain 26695.^20^ *HP0043* gene codes for the bifunctional isomerase RfbM, which is involved in the fucose biosynthesis pathway. Fucose is a key component in the terminal and central trio region of LPS O-antigen in *H. pylori*. RfbM not only can convert fructose-6P to mannose-6P but also mannose-1P to GDP-mannose. HP0208 is predicted to be a glycosyltransferase that is homologous to *S. typhimurium* WaaJ, an enzyme involved in the LPS core biosynthesis. *HP0360* gene codes for the epimerase GalE, which can convert UDP-glucose to UDP-galactose. Galactose is the backbone sugar of LPS O-antigen repeat units in *H. pylori* and forms a poly *N*-acetyllactosamine (LacNAc) with GlcNAc. Both *HP0379* and *HP0651* genes code for fucose transferases (FutA and FutB, respectively) for Lewis antigens decorated on the terminus of LPS O-antigen in *H. pylori. HP0826* gene codes for a galactose transferase, which can transfer UDP-galactose to the O-antigen region of LPS and generate a complete LacNAc (Supplementary Table S1). The molecular sizes of these adhesins in the LPS biosynthesis-related knockout mutants *HP0043, HP0360*, and *HP0826* were smaller than those in the wild-type strain 26695 (Figure 1c). The molecular sizes of adhesins recovered fully after the *HP0858* gene was complemented into the *HP0858* knockout mutant, while the size recovery observed after *HP0859* complementation was less significant (Figure 1d). Furthermore, after treatment with trifluoromethanesulfonic acid (TFMS) for protein deglycosylation, the molecular sizes of all tested adhesins in the wild-type strain 26695 decreased as well, with the corresponding sizes similar to those in the *HP0858* knockout mutant (Figure 1e).

According to recent studies, some proteins in *H. pylori* could be metabolically labeled with azide-containing sugar tetraacetylated N-azidoacetylglucosamine (Ac_4_GlcNAz) which is a biorthogonal substitute for the endogenous amino sugar N-acetylglucosamine (GlcNAc), a component of *H. pylori* LPS.^21,22^ To further elaborate adhesin glycosylation in *H. pylori*, an approach based on the combination of metabolic labeling and click chemistry was also employed in this study. *H. pylori* 26695 wild-type strain was first metabolically labeled with an azide-containing sugar, Ac_4_GlcNAz, and the derived total cell lysates and outer membrane samples were then treated with a probe containing the alkyne-biotin moiety to perform a click reaction between an azide and an alkyne, followed by immunoblotting analysis with streptavidin-HRP and anti-BabA antibody simultaneously. As shown in figure 1f, while the total cell lysates from metabolically-labeled bacteria were used for testing, no azide-labeled signals were observed in lysates exempting from executing the click reaction with an alkyne-biotin probe (Click-iT biotin), whereas the addition of alkyne-biotin probe treatment generated a robust azide-labeled signal. Ponceau S staining analysis confirmed the equivalent loading of protein levels (figure 1f). The above data not only demonstrate the exceptional specificity of the test but also agree that *H. pylori* can employ Ac_4_GlcNAz as a metabolic substrate and many glycoproteins existing in this bacterium can be azide-labeled. Encouragingly, when an anti-BabA antibody was applied to identify BabA, the obtained fluorescence signal mostly overlapped with one of the azide-labeled signals, indicating that BabA is incorporated with azido-containing moieties after metabolic labeling with Ac_4_GlcNAz. Furthermore, to assess the likelihood that the azide moiety is also incorporated into LPS, a component frequently present in the total cell lysates of Gram-negative bacteria, proteinase K digestion was conducted on the azide-labeled, click chemistry-performed sample to remove proteins prior to immunoblotting detection. Ponceau S staining analysis verified that protein digestion was nearly complete, and immunoblotting analysis showed that no azide-labeled signals were observed after the proteinase K treatment (figure 1f), implying that Ac_4_GlcNAz is metabolically incorporated into the cellular proteins but not the LPS of *H. pylori* under current experimental conditions. This result supports the fact that what is observed in this study is truly adhesin glycosylation but not non-covalent association of the components of LPS to *H. pylori* adhesins. Similar conclusions were also drawn from the tests with azide-labeled outer membrane samples (Figure 1g). In this set of experiments, the azide-dependency of tests was also confirmed by utilizing the azide-free controls.

Even with the above evidences of adhesin glycosylation, however, no obvious difference in protein electrophoretic migration was observed after treatment with Peptide-*N*-Glycosidase F (PNGase F) for the N-linked enzymatic deglycosylation analysis, β-elimination for the O-linked chemical deglycosylation analysis, or α1-3,4 fucosidase and α1-2 fucosidase treatments for the removal of fucose residues (Supplementary Figure S2A-C). Intriguingly, while the outer membrane protein samples were pretreated with α1-3,4 fucosidase prior to treatment with PNGase F, a band with an apparent molecular mass down-shifted to a size similar to that in the *HP0859* knockout mutant was observed for BabA (Supplementary Figure S2D), suggesting the possibility that fucose residues may interfere with the action of PNGase F in the removal of N-glycans. The above findings, collectively, implied that key adhesins in *H. pylori* were glycosylated by enzymes involved in LPS biosynthesis, but which type of protein glycosylation is responsible for the glycan modification of key adhesins in *H. pylori* remains inconclusive and deserves further investigation.

### Key adhesins in *H.* *pylori* display a two-step upshift glycosylation pattern

**Figure 2.**
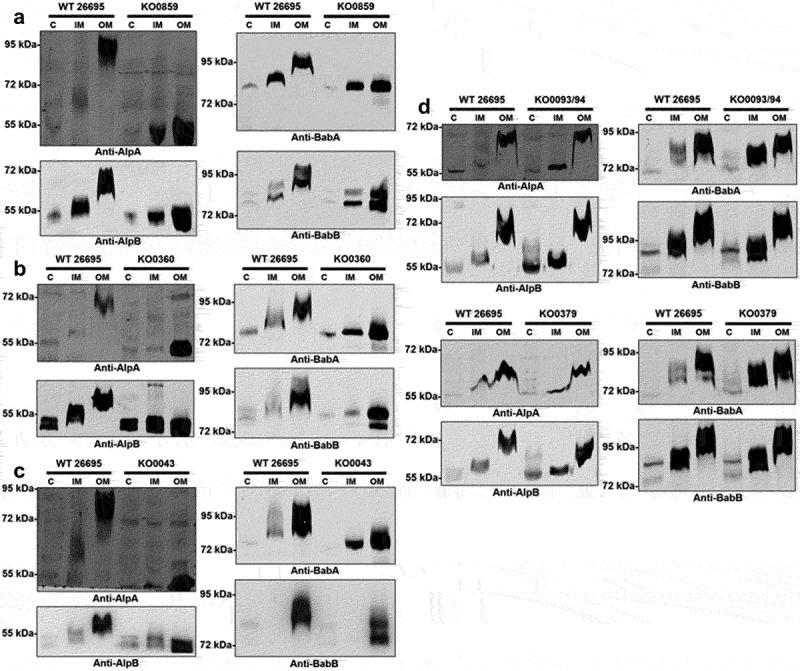
Adhesin glycosylation displays a two-step molecular weight upshift pattern in *H. pylori*. Electrophoretic migration of the tested adhesins. Protein samples from each subcellular fraction of various *H. pylori* strains were analyzed using 12% SDS–PAGE gels for AlpA/B separation or 10% SDS–PAGE gels for BabA/B separation. The adhesin electrophoretic migration pattern of (a) the *HP0859*, (b) *HP0360*, (c) *HP0043*, (d) *HP0093/94* and *HP0379* knockout mutants was compared with that of the wild-type strain 26695 by performing immunoblotting with antibodies against AlpA, AlpB, BabA and BabB. Protein samples were isolated from the cytoplasm (C), inner membrane (IM) and outer membrane (OM) of bacteria. WT, wild-type strain; KO, knockout mutant.

Subcellular fractionation was applied, and protein samples from the cytoplasm, inner membrane, and outer membrane were collected for immunoblotting to obtain an in-depth understanding of the process of adhesin glycosylation in *H. pylori*. The molecular sizes of all tested adhesins AlpA, AlpB, BabA, and BabB displayed a two-step upshift pattern from the cytoplasm to the inner membrane and from the inner membrane to the outer membrane in the wild-type strain 26695 (Figure 2). However, this molecular size upshift pattern was not observed in the *HP0859, HP0360*, and *HP0043* (for heptose, galactose, and fucose biosynthesis, respectively, Supplementary Table S1) knockout mutants (Figure 2a-c), suggesting that adhesin glycosylation occurs during protein transportation from the inner membrane to the outer membrane, and the glycans on these key adhesins may be comprised of heptose, galactose, fucose, and/or derivatives of these sugars. However, other possibilities still cannot be excluded, such as the molecular weight change of adhesins observed from interfering with the biosynthesis of these sugars could be actually due to their impact on the alteration of carbohydrate metabolism flux. Further studies are still required to validate the above claim concerning the monosaccharide components of key adhesins in *H. pylori*.

The proteins encoded by the *HP0093/94* and *HP0379* genes are also enzymes involved in LPS biosynthesis (*HP0093/94* gene codes for another fucose transferase (FutC) for Lewis antigen decoration on the terminus of LPS O-antigen in *H. pylori*; Supplementary Table S1). The data showed similar migration patterns of adhesins in different subcellular fractions from the *HP0093/94* knockout mutant and the *HP0379* knockout mutant to those from the wild-type strain 26695 (Figure 2d). However, it is worth noting that some protein bands in the inner membrane fraction from the fucose biosynthesis-related knockout mutants migrated slightly faster than those from the wild-type strain 26695. The above findings suggest it is possible that a loss of a single fucose residue might yield only a minor difference in adhesin migration in SDS-PAGE that would be difficult to clearly resolve under the experimental conditions currently used.

### Adhesin glycosylation is also present in other sequenced *H.* *pylori* strains and clinically isolated strains

To determine the universality of adhesin glycosylation in *H. pylori*, sequenced strains G27, J99, and NCTC 11637, and clinically isolated strains 13209, 14635, and 14637 (Supplementary Table S2) were further chosen to assess the electrophoretic mobility of the key adhesins in different subcellular fractions (Supplementary Figure S3A-F). Although the patterns of adhesin migration displayed a certain degree of difference among the strains tested compared to those of the wild-type strain 26695, they all showed a molecular size upshift pattern starting from the transient presence in the inner membrane or at least in their final destination of the outer membrane. After treatment with TFMS for protein deglycosylation, the molecular sizes of most tested adhesins from these strains decreased, similar to those in the wild-type strain 26695 subjected to the same TFMS treatment (Supplementary Figure S3G). Based on these results, adhesin glycosylation not only exists in the standard strain 26695 but is also present in other strains of *H. pylori*, and glycosylation seems to be an important adhesin modification due to the prevalence among strains of various origins.

### The enzymes required for adhesin glycosylation in *H.* *pylori* are also involved in LPS O-antigen biosynthesis

Increasing evidence has shown that some gram-negative bacteria utilize enzymes associated with LPS O-antigen biosynthesis for glycan construction and transfer, which are critical for later posttranslational protein glycosylation.^23,24^ A previous research also indicated that Wzk, a flippase involved in LPS O-antigen transport *in H. pylori*, can substitute the function of the enzyme PglK, a heptasaccharide flippase engaged in the protein glycosylation pathway in *Campylobacter jejuni*.^25^ Based on the existing examples and our accumulating data, we therefore propose that the enzymes involved in adhesin glycosylation may also be involved in LPS O-antigen biosynthesis in *H. pylori*.

**Figure 3.**
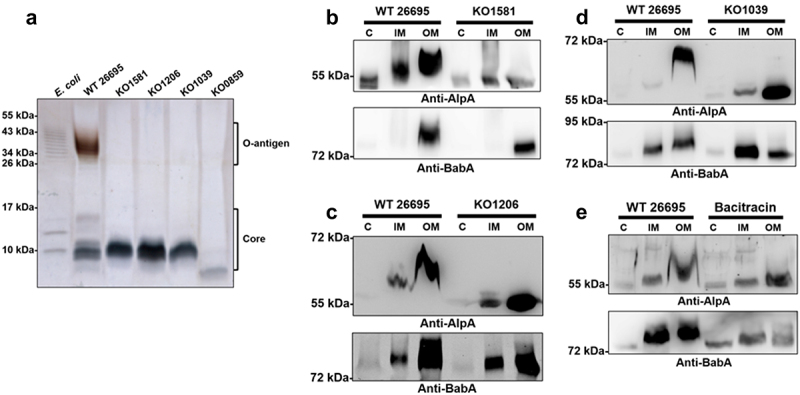
Disruption of the LPS O-antigen biosynthetic pathway in the wild-type strain 26695 drastically alters the electrophoretic migration patterns of key adhesins. (a) The successful generation of LPS O-antigen biosynthesis-disrupted mutants was confirmed by silver staining to reveal the LPS profiles of mutants. LPS samples were isolated from the total bacterial cell lysate. LPS samples from the wild-type strain 26695 and the *HP0859* knockout mutant were applied as the controls for comparison. (b-d) The electrophoretic migration patterns of the tested adhesins from (b) *HP1581 (WecA)*, (c) *HP1206 (Wzk)* and (d) *HP1039 (WaaL)* knockout mutants were compared to those of the wild-type strain 26695 by performing immunoblotting with antibodies against AlpA and BabA. (e) The wild-type strain 26695 treated with or without bacitracin was subjected to subcellular fractionation, and the adhesin migration patterns were compared using immunoblotting with antibodies against AlpA and BabA. Protein samples isolated from the cytoplasm (C), inner membrane (IM) and outer membrane (OM) of bacteria were separated on 12% SDS–PAGE gels for AlpA separation or 10% SDS–PAGE gels for BabA. WT, wild-type strain; KO, knockout mutant.

Gene-disrupted mutants targeting the LPS O-antigen assembly and ligation enzymes WecA, Wzk, and WaaL were generated from the wild-type strains 26695 and G27 and confirmed by LPS silver staining analysis to verify this hypothesis (Figure 3a and Supplementary Figure S4A). Compared to the tested adhesins in the wild-type strains 26695 and G27, the two-step molecular weight upshift glycosylation patterns all disappeared in the corresponding knockout mutants, suggesting that these LPS O-antigen biosynthesis-related enzymes are necessary for the posttranslational modification of the tested adhesins in *H. pylori* (Figure 3b-d and Supplementary Figure S4B-D). The glycans required for O-antigen biosynthesis are assembled onto and transferred by an inner membrane lipid carrier, undecaprenyl phosphate (UndP).^26^ The synthetic and recycling pathway of UndP has been reported to be inhibited by an antibiotic, bacitracin.^26^ After bacitracin treatment, the molecular size upshift patterns of the adhesins also disappeared, thus further confirming that the three O-antigen biosynthesis-related enzymes tested here indeed participate in glycan transfer for adhesin glycosylation in *H. pylori* (Figure 3e).

**Figure 4.**
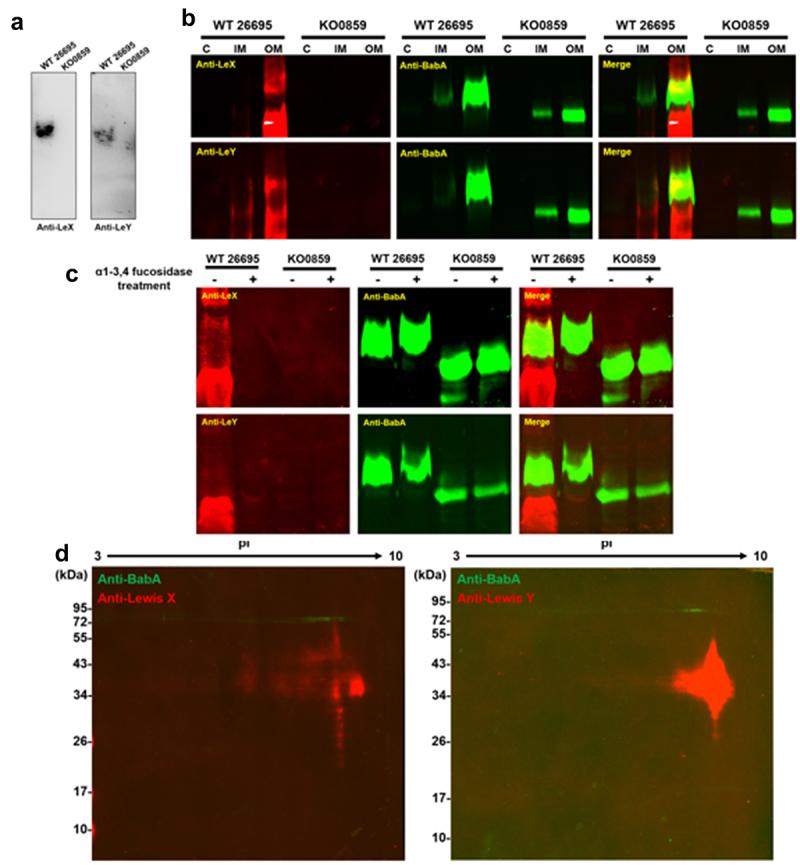
BabA in the wild-type strain 26695 is not modified with Lewis X and Lewis Y antigens. (a) Decoration of Lewis X (LeX) and Lewis Y (LeY) antigens on the O-antigen of the wild-type strain 26695 was detected and confirmed using immunoblotting with anti-Lewis X and anti-Lewis Y antibodies, respectively. LPS samples were isolated from the total bacterial cell lysate and subjected to analysis. Evaluation of overlapping signals for BabA with Lewis X and Lewis Y antigens by (b) immunoblotting analysis, (c) after α1-3,4 fucosidase treatment, or (d) 2D-PAGE. The bands of BabA, Lewis X antigen, and Lewis Y antigen were detected using immunoblotting with anti-BabA, anti-Lewis X and anti-Lewis Y antibodies and visualized using the fluorescence gel scan method (red: 700 nm emission, Lewis X antigen and Lewis Y antigen; green: 800 nm emission, BabA). Protein samples isolated from the cytoplasm (C), inner membrane (IM) and outer membrane (OM) of the wild-type strain 26695 and the *HP0859* knockout mutant were separated on 10% SDS–PAGE gels or 12.5% SDS–PAGE for BabA. WT, wild-type strain; KO, knockout mutant.

Due to the connection between LPS O-antigen biosynthesis and the adhesin glycosylation pathway, we speculate that the glycan moiety in these adhesins may also be similar to that in LPS O-antigen, as reported in some bacteria.^23,27^ The terminus of LPS O-antigen in the wild-type strains 26695 and G27 has been reported to be modified by fucose residues and form special glycan structures called Lewis X and/or Lewis Y antigens that mimic the surface glycans of host epithelial cells for evading immune surveillance. We first confirmed the presence of Lewis antigens X and Y in the LPS structure of the wild-type strain 26695 to reveal the details of the glycan moiety in the tested adhesins, but these two glycan moieties were absent in the *HP0859* knockout mutant (Figure 4a). Next, immunoblotting with a fluorescence gel scan method was applied to the subcellular fractions to simultaneously detect the signals for BabA, Lewis X, and Lewis Y antigens. As shown in Figure 4b, the signals for BabA, Lewis X, and Lewis Y were only simultaneously detected in the inner membrane and the outer membrane of the wild-type strain 26695. Once the obtained fluorescence signals were overlapped, the BabA signal partially overlapped with that of the Lewis X and Lewis Y antigens (Figure 4b). Interestingly, this signal overlap phenomenon disappeared in the *HP0859* knockout mutant. Similar findings were also observed while testing the overlapping signals of AlpA and AlpB with the Lewis Y antigen in the wild-type strain G27 (Supplementary Figure S4E and F). However, the results from the α1-3,4 fucosidase treatment showed that the molecular weight of BabA in wild-type strain 26695 did not change after the treatment, and no overlapping signal between BabA and Lewis X/Y antigens was observed (Figure 4c). To further confirm the above findings, we also conducted the 2D-PAGE followed by immunoblotting and the results showed that the signals of both Lewis X and Y antigens were not overlapped with those of the BabA protein (Figure 4d). Altogether, it is suggested that the key adhesins in *H. pylori* can be modified by glycan moieties that are also present in LPS; however, based on our findings, the Lewis X and Y epitopes are not included in the adhesin modification. The overlapping signals observed between adhesins and Lewis X and Y antigens may be simply due to the co-migration of BabA with the Lewis-reactive LPS present in the outer membrane fraction.

### The protease resistance, protein stability, and binding ability of key adhesins are strongly supported by the glycan modification

**Figure 5.**
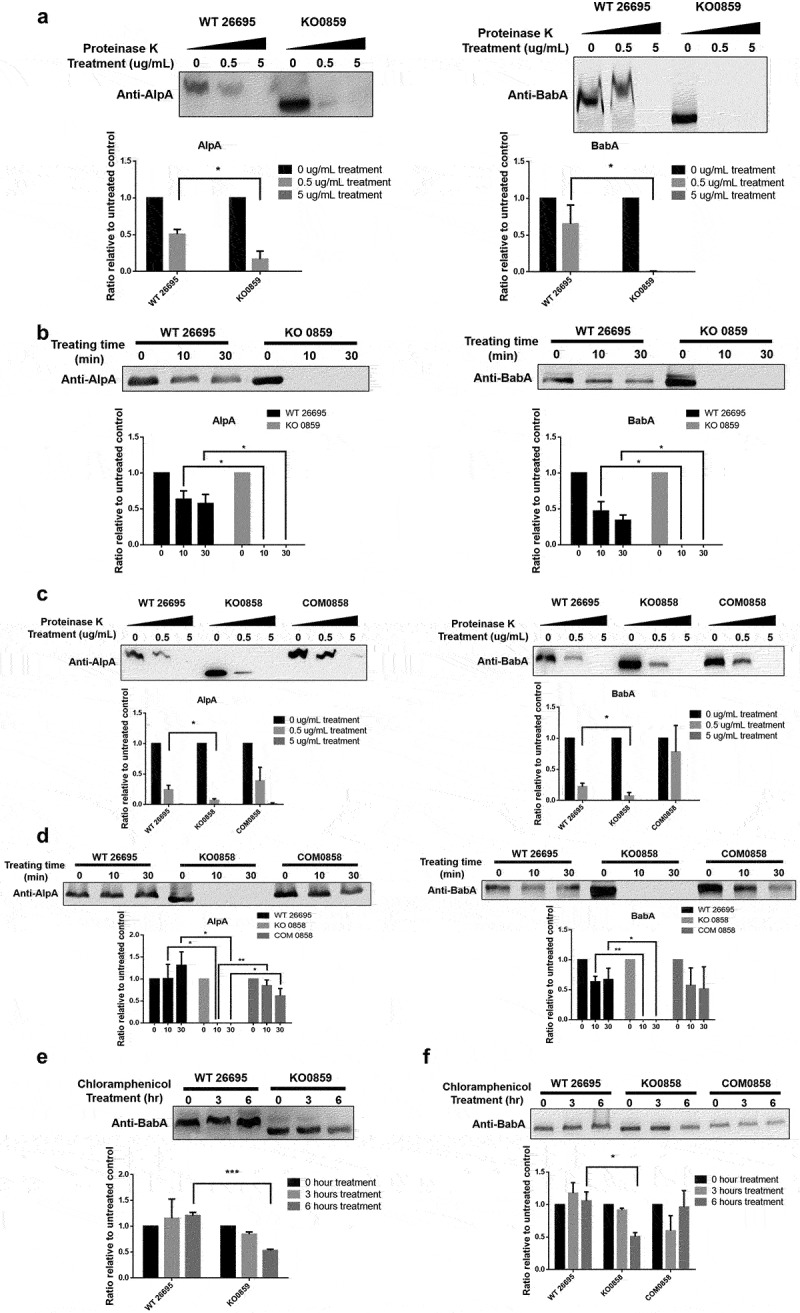
Protease resistance and protein stability of key *H. pylori* adhesins are strongly supported by glycan modification. (a and c) The amounts of AlpA and BabA in the outer membrane samples collected from various strains of *H. pylori* treated with different concentrations of proteinase K (0, 0.5 and 5 μg/mL) were compared using immunoblotting with anti-AlpA and anti-BabA antibodies. (b and d) The amounts of AlpA and BabA in the outer membrane samples collected from various strains of *H. pylori* treated with 0.5 μg/mL proteinase K in a time course analysis (0, 10 and 30 minutes) were compared using immunoblotting with anti-AlpA and anti-BabA antibodies. (e and f) The amount of BabA in the outer membrane samples collected from various strains of *H. pylori* incubated with 500 μg/mL chloramphenicol for different periods (0, 3 and 6 h) was compared using immunoblotting with an anti-BabA antibody. The experiments were repeated three times, and one representative blot (upper panels) and the densitometry analysis (lower panels) are shown (unpaired, two-tailed Student’s t test; n = 3; *p < .05, **p < .01 and ***p < .001). Quantification was performed using ImageJ (1.53e) software. Protein samples from the outer membrane fraction were separated on 12% SDS–PAGE gels for AlpA or 10% SDS–PAGE gels for BabA. WT, wild-type strain; KO, knockout mutant; COM, knockout complementary mutant.

To assess the effect of disrupting adhesin glycosylation on *H. pylori*, proteinase K treatment was first used to evaluate the sensitivity of adhesins toward protease digestion. As shown in Figure 5a,c, treatment with 0.5 μg/mL proteinase K alone decreased the amounts of the tested adhesins, BabA, and AlpA, in the *HP0859* and *HP0858* knockout mutants much more remarkably than those from the wild-type strain 26695. In Figure 5b,d, an even more significant difference can be observed when the proteinase K treatment was performed as a time-dependent analysis. Furthermore, chloramphenicol was added to *H. pylori* cultures to block nascent protein synthesis and probe the effect of protein glycosylation on adhesin stability. In these two LPS biosynthesis-disrupted mutants, BabA levels were reduced significantly after 6 h of antibiotic treatment, while the amount of intact BabA in the wild-type strain 26695 remained similar in all treated time periods (Figure 5e,f). The *HP0858* knockout complementary mutant was also investigated in these three assays, and the corresponding results were comparable to those of the wild-type strain 26695, thus excluding the concern of polar effects in the aforementioned findings (Figure 5). These data suggest that for *H. pylori*, adhesin glycosylation exerts a mild-to-moderate effect not only on its resistance to protease digestion but also on the maintenance of adhesin stability.

**Figure 6.**
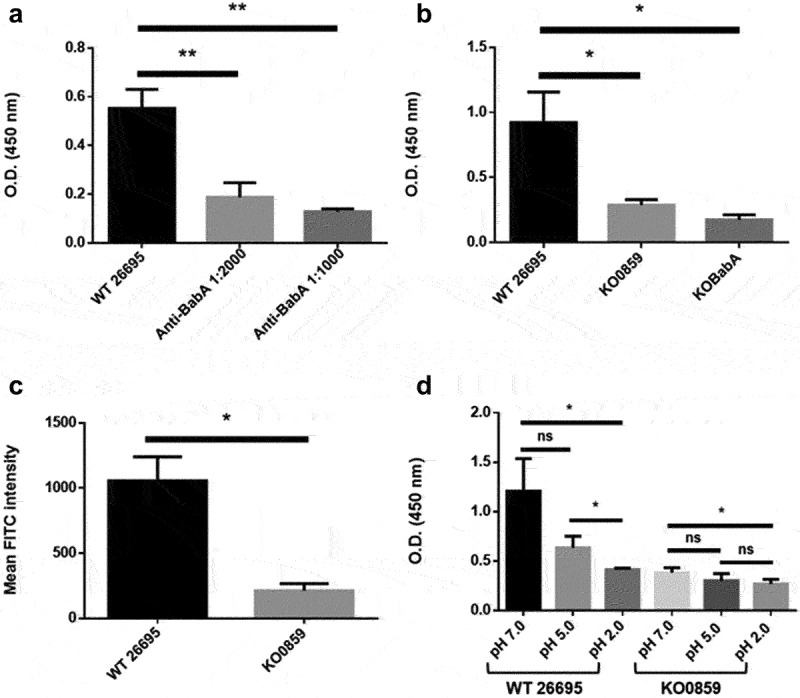
BabA binding is affected by protein glycosylation. (a) Different dilution ratios of BabA-specific antibody were applied in the Lewis B binding competition assay, and the effect of antibody addition on bacterial binding was compared using an ELISA. Bacterial binding to the BabA receptor, Lewis B antigen, was compared and evaluated for the wild-type strain 26695 and various mutants using (b) ELISA and (c) flow cytometry analysis. Data are presented as the average values derived from triplicate trials with statistical analysis (unpaired, two-tailed Student’s t test; *p < .05 and **p < .001). (d) The pH-dependent binding flexibility between BabA and the Lewis B-BSA complex was significantly reduced in the glycosylation-deficient *HP0859* knockout mutant. The wild-type strain 26695 and the *HP0859* knockout mutant cultured under different pH conditions were subjected to ELISA for Lewis B binding (pH 7.0, pH 5.0 and pH 2.0). Data are presented as the average OD_450_ values derived from triplicate trials with statistical analysis (unpaired, two-tailed Student’s t test; *p < .05). WT, wild-type strain; KO, knockout mutant; ns, not significant.

Next, the effect of adhesin glycosylation on its binding ability to the host cell surface receptor was tested. BabA and its host receptor Lewis B antigen were chosen for subsequent tests. An antibody competition assay was first conducted to confirm that the binding results were mostly attributed to the interaction between BabA and the Lewis B antigen (Figure 6a). Then, the interaction of this ligand–receptor pair was evaluated in different *H. pylori* strains. The binding ability of BabA in the wild-type strain 26695 was significantly higher than that in the heptose-deficient *HP0859* and the negative control *babA* knockout mutant (Figure 6b). The binding between BabA and the Lewis B antigen was also tested using flow cytometry, and a similar trend was observed (Figure 6c). Taken together, glycosylation is crucial for maintaining the ability of BabA to bind its ligand, Lewis B antigen.

The BabA binding ability is acid-sensitive, and as the pH decreases gradually, the ability of BabA to adhere to its receptor Lewis B antigen is also reduced.^28^ To explore whether adhesin glycosylation affects the acid sensitivity of BabA binding, a binding assay was conducted under different pH conditions to assess the ability of BabA to bind to the Lewis B antigen (Figure 6d). When the pH was maintained at 7.0, the binding ability of BabA in the wild-type strain 26695 was significantly higher than that in the *HP0859* knockout mutant. However, this binding difference was gradually reduced as the pH decreased. This finding suggests that the glycan modification of BabA may support the protein structure of the adhesin and thus promote the adherence of *H. pylori* to gastric cells; this supporting role for BabA binding is generally lost when bacteria are located in a low pH environment, such as gastric lumen.

### Heterologously expressed BabA cannot be modified by glycans

Mass spectrometry analysis is the most commonly used approach to determine the glycan components and structure of a glycoprotein. We attempt to explore the composition and structure of glycans in these key adhesins in great detail by heterologously expressing one of the adhesins, BabA, in *E. coli* and purifying a sufficient amount of this glycoprotein for a subsequent glycan analysis. However, the results showed that exogenously expressed BabA was not modified by glycans, and the molecular size upshift pattern from the cytoplasmic fraction to the outer membrane fraction was lost (Supplementary Figure S5). This unsuccessful result may possibly be due to the specificity of enzymes engaged in the protein glycosylation process. Unlike the general glycosylation systems described for *C. jejuni*, most of the genes encoding enzymes shared by LPS biosynthesis and adhesin glycosylation pathways are scattered throughout the genome of *H. pylori*. These facts imply that fully glycosylated BabA can only be obtained through direct expression in and purification from *H. pylori*. We then attempt to apply several approaches to obtain adhesins, including BabA and AlpA, directly from *H. pylori*, but only very limited success has been achieved thus far (data not shown), probably due to a lack of a reliable and practical system for protein expression in *H. pylori* or because of the physical properties of membrane proteins that are invariably difficult to express and purify. Therefore, to obtain the details of the glycan structure of BabA, further research directly in *H. pylori* are still in need.

## Discussion

For decades, researchers have believed that protein glycosylation is a complex post-translational modification that only exists in eukaryotic organisms. However, increasing evidence convincingly shows that prokaryotes also possess protein glycosylation systems with either N- or O-linkages. According to previous studies, *H. pylori* potentially possesses more than one protein glycosylation system as well.^21,29^ The flagellin protein in *H. pylori* is modified by pseudaminic acid, and the glycan in the flagellin protein is important for the function of flagella.^30^ In another study, a new glycan labeling method coupled with mass spectrometry analysis was applied, and many glycoproteins in *H. pylori* were identified.^21,22,31^ The subcellular fractions of the cytoplasm, inner membrane, periplasm, and outer membrane of *H. pylori* contained many glycoproteins and 125 proteins, including BabA, were identified through metabolic labeling of glycans. In this study, we used genetic and biochemical approaches to show that the key adhesins AlpA/B and BabA/B in *H. pylori* are indeed modified with glycans.

By performing subcellular fractionation and immunoblotting, we identified that the molecular sizes of all tested adhesins displayed a two-step upshift pattern from the cytoplasm to the outer membrane, indicating that the glycosylation process of these adhesins may occur during or immediately after protein transport across the inner membrane. Studies have suggested that protein N-linked glycosylation in bacteria occurs before complete folding of substrate proteins and is a co-translocation process during protein translocation across the inner membrane.^32^ The gradual upshift patterns of adhesins observed in this study imply that they are simultaneously transported to the periplasm and modified by glycans. Therefore, a reasonable hypothesis is that adhesins present in the outer membrane fraction are the complete glycosylated form of adhesins, while those observed in the inner membrane fraction are still undergoing the glycosylation process and considered semi-glycosylated adhesins. However, another possible interpretation that cannot be completely excluded is the presence of a second glycosylation system in the periplasm that is yet to be identified and will complete the glycosylation process for these adhesins. Further studies are required to differentiate the two assumptions described above and reveal the detailed mechanism of adhesin glycosylation in *H. pylori*.

Our findings in this study disclose an interesting fact that, for those LPS biosynthesis-related enzymes indeed involved in adhesin glycosylation, the molecular sizes of these key adhesins in the inner membrane and the outer membrane of the corresponding gene-disrupted mutants were all reduced to similar sizes as shown in the cytoplasm and there is no difference in the electrophoretic migration patterns among these knockout mutants including *HP0859, HP0360*, and *HP0043*. In addition, for the outer membrane samples, the degrees of molecular weight reduction in these tested adhesins were similar among the glycosylation-deficient mutants and the wild-type strain 26695 treated with the deglycosylation reagent TFMS to remove all glycan types. Based on these results, the component sugar residues are not likely transferred individually and progressively to the acceptor adhesins. Instead, the intact sugar moiety has to be assembled first by the stepwise addition of monosaccharides onto a carrier and then *en bloc* transferred to the adhesins in *H. pylori*. Once one of the sugar components in the glycan moiety is missing, the glycan moiety is not synthesized properly, and thus the adhesin glycosylation process is abolished completely.

*Campylobacter* species,^33–35^ *Neisseria* species,^36–38^ and *Burkholderia* species^39^ have a so-called general glycosylation system in which the enzymes involved in biosynthesis and transfer of the glycan moiety are chromosomally encoded within a gene locus. In the current study, *H. pylori* seems to represent another bacterium that possesses a general protein glycosylation system, at least for outer membrane adhesins. A previous study demonstrated that Wzk, the O-antigen flippase in *H. pylori*, can substitute for *C. jejuni* PglK to translocate UndPP-linked glycans and participate in the protein glycosylation process of *C. jejuni*.^25^ The enzymes involved in O-antigen biosynthesis were found to share with those for pilin and flagellin glycosylation in *Pseudomonas aeruginosa*.^23,24^ Recently, a study also showed that protein glycosylation in *H. pylori* indeed proceeds via a lipid carrier-mediated pathway that overlaps with the process of LPS O-antigen biosynthesis.^40^ In that research, HPG27_1518, a homolog of HP1581 (WecA), was identified to be involved in the protein glycosylation pathway in the *H. pylori* G27 strain. In our current study, the *HP1581, HP1206*, and *HP1039* knockout mutants were generated from both the 26695 and G27 strains, and the samples from subcellular fractionation were subjected to immunoblotting analysis. Our findings reveal that the key adhesins from most of the generated LPS biosynthesis-disrupted mutants have faster electrophoretic mobility than those from the parental strains 26695 and G27, suggesting that some of the LPS biosynthesis-related enzymes and those associated with O-antigen biosynthesis and translocation in particular indeed participate in the adhesin glycosylation process. Additionally, at least part of the glycan moiety used in the modification process might be similar to that present in LPS. Therefore, our findings strongly suggest that at least some of the enzymes required for adhesin glycosylation are involved in LPS O-antigen biosynthesis in *H. pylori*, consistent with the recent findings described above.^40^

Protein glycosylation in bacteria can modulate the physical and functional properties of a protein, such as susceptibility to protease degradation,^41^ protein stability,^42^ adherence,^43^ and bacterial fitness.^44^ In the present study, glycan modification of adhesins BabA and AlpA provided a certain degree of protection against proteinase K digestion. In addition, the amount of BabA in the wild-type strain 26695 remained steady during the course of antibiotic chloramphenicol treatment, while the amount of this protein in the glycosylation-deficient mutants decreased with increasing treatment time, suggesting that the stability of adhesin BabA is increased by protein glycosylation. To further explore the effect of glycan modification on the BabA function, ELISA binding assays and flow cytometry analyses were applied to evaluate the interaction between BabA and its receptor Lewis B antigen. The results of both experiments supported the conclusion that glycosylation of BabA can promote its binding to the Lewis B antigen, thus strengthening the adherent function of BabA.

However, one conundrum must be addressed. According to a recent study,^10^ no glycan moiety is present at the BabA/Lewis B antigen binding interface when the BabA protein is heterologously expressed in *E. coli*. The essential components of BabA binding are the formation of the correct hydrogen bonds between the BabA binding domain and Lewis B antigen and the formation of disulfide bonds within the BabA binding domain to maintain the domain structure. BabA binding is abolished by treatment with a redox-active drug, N-acetylcysteine, which breaks the disulfide bonds of BabA through a reduction reaction. Based on these findings, a stable structure of the BabA binding domain is important to maintain its binding ability. Nevertheless, the stability of the BabA structure is easily damaged since *H. pylori* lives in an extreme environment enriched in protons and proteases. The protein structure may be easily denatured to adopt a dysfunctional form in an acidic environment, and proteins exposed to such harsh conditions may be even more vulnerable to degradation by gastric proteases. Studies have indeed shown that the post-translational modification of a protein exerts a significant effect on the protein conformation. It has been suggested that protein modification may induce alterations in partial or even overall protein structure and change the number of hydrogen bonds and salt bridges in the protein structure.^45^ The study also showed that protein conformation and dynamics are affected by protein glycosylation.^46^ The role of glycans in a protein is similar to glue, which can reduce protein dynamics and stabilize the protein structure. Therefore, we suggest that BabA glycosylation may not directly participate in the interaction between BabA and the Lewis B antigen; instead, the glycosylation of this adhesin may maintain or reinforce the protein conformation and indirectly enhance its binding ability. The same suggestion may also apply to at least other key adhesins tested in this study, which were all shown to be modified by glycans. This assumption may also explain why LPS biosynthesis-related, glycosylation-deficient mutations exert a more significant effect on *H. pylori* adhesion than the BabA-disrupted mutation itself. The enzymes shared by LPS biosynthesis and adhesin glycosylation work like a power switch, and all light bulbs connected to it will be simultaneously turned off if this power switch is not functioning.

**Figure 7.**
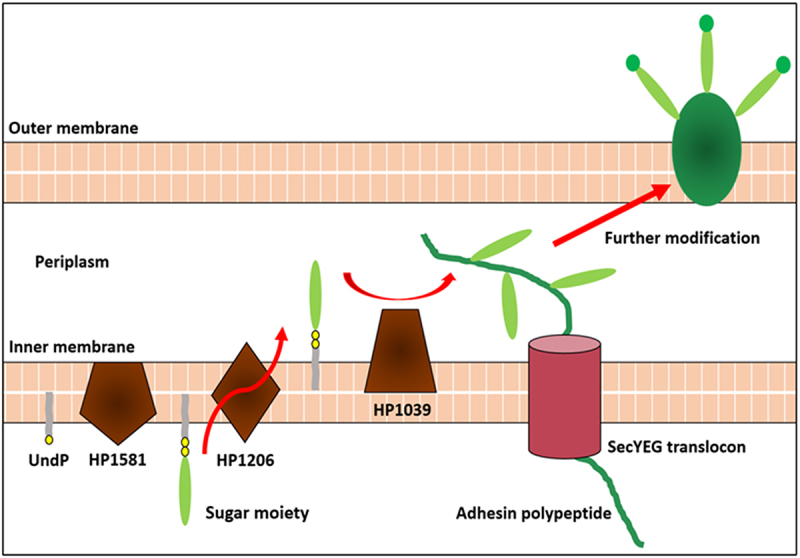
The proposed model of adhesin glycosylation in *H. pylori*. Some of the enzymes involved in adhesin glycosylation in *H. pylori* are also involved in LPS biosynthesis. Sugar moieties are synthesized by O-antigen-included LPS biosynthesis enzymes and simultaneously added to the adhesin polypeptide while the adhesin polypeptide is being translocated to the periplasm. Fully glycosylated adhesins will be successfully folded and finally embedded in the outer membrane of bacteria. The glycan modification may enhance adhesin binding ability by stabilizing the adhesin structure and thus promoting the fitness and virulence of *H. pylori.*

In summary, we propose the model described below for adhesin glycosylation in *H. pylori* (Figure 7). The glycan moiety used for adhesin glycosylation is synthesized and transferred by enzymes involved in O-antigen-included LPS biosynthesis. The component sugar residues are initiated by WecA and then successively added to the lipid carrier UndPP on the cytoplasmic face of the inner membrane. After the glycan moiety is fully constructed, it will be translocated across the inner membrane to the periplasm by the O-antigen flippase Wzk. When the adhesin polypeptide is transferred to the periplasm, the glycan moiety will be *en bloc* ligated to the polypeptide synchronously by the O-antigen ligase WaaL. Finally, the adhesin will be successfully folded and integrated into the outer membrane. The modification of glycans may increase the binding ability and protein stability by stabilizing the adhesin structure. Therefore, this newly identified protein glycosylation system provides a series of potential drug targets for therapeutic invention to combat *H. pylori* infection, and further research is worthwhile.

## Materials and methods

### Bacterial strains and growth conditions

The details of the *H. pylori* strains used in this study are listed in Supplementary Table S2. The sequenced strains used were *H. pylori* strain 26695 (ATCC 700392),^47^ strain G27,^48,49^ strain J99 (ATCC 700824),^50^ and strain NCTC 11637 (ATCC 43504).^51^ The clinically isolated strains were strain 13209, strain 14635, and strain 14637, which were provided by Dr D.C. Wu’s laboratory from Kaohsiung Medical University Hospital. Bacteria were cultured in Brucella broth (Gibco, Grand Island, NE, USA) with 10% fetal bovine serum (Biological Industries, Kibbutz Beit-Haemek, Israel) and 1% IsoVitaleX (Taiwan Prepared Media, Taipei, Taiwan) in a sealed jar under microaerophilic conditions (5% O_2_, 10% CO_2_, and 85% N_2_) with shaking at 120 rpm in a horizontal-type incubator. (LM-510RD, Yihder Co., New Taipei, Taiwan) at 37°C for 48 h.

### Construction of gene knockout mutants of *H.* *pylori*

The construction of gene-disrupted mutations in *H. pylori* was conducted as described previously.^16^ Briefly, the genes *HP0043, HP0093/94, HP0208, HP0360, HP0379, HP0651, HP0826, HP0857, HP0858, HP0859, HP1039, HP1206, HP1581*, and *babA (HP1243)* from the *H. pylori* wild-type strain 26695, and *GHP_389 (waaL), GHP_1153 (wzk)*, and *GHP_1518 (wecA)* from the *H. pylori* wild-type strain G27 were individually amplified using the primers listed in Supplementary Table S3 and cloned into the pGEMT vector (Promega, Madison, WI, USA). A vector containing an interrupted gene of interest was constructed by introducing a *Bam*HI restriction enzyme site into the target gene sequence in the pGEMT vector, and a chloramphenicol resistance cassette (all excluded *HP1581*) or a kanamycin resistance cassette (for *HP1581*) was then inserted into the *Bam*HI restriction site. The resulting vector was transformed into wild-type strains 26695 and G27 through natural transformation, and gene interruption was autonomously completed by homologous recombination. The successful insertion of the resistance cassette was confirmed by polymerase chain reaction (PCR) analysis.

### Subcellular fractionation

*H. pylori* cultured to the logarithmic phase was pelleted (4,000*×*g, 10 minutes, 4°C) and washed three times with phosphate-buffered saline (PBS). The pellet was resuspended in 20 mM Tris-HCl (pH 7.4) and lysed by sonication (30% amplitude, 10 minutes in total with a five-second interval between each cycle) (Thermo Fisher Scientific, Waltham, MA, USA). The cell debris was removed by centrifugation (10,000*×*g, 10 minutes), and the supernatant was collected and centrifuged for 45 minutes at 50,000*×*g. After centrifugation, the resulting supernatant was collected as the cytoplasmic fraction, and the pellet was the total membrane fraction, which contained the inner membrane and the outer membrane of bacteria. The total membrane fraction was resuspended in 20 mM Tris-HCl containing 2% *N*-lauroyl sarcosine (Sigma–Aldrich, St Louis, MO, USA), which emulsified inner membrane lipids and solubilized inner membrane proteins. The resulting solution was centrifuged for 45 minutes at 50,000*×*g, and the obtained supernatant was collected as the inner membrane fraction. Finally, the pellet, which represents the outer membrane fraction, was resuspended in 20 mM Tris-HCl. The final protein samples from the cytoplasmic and inner membrane fractions were concentrated by acetone precipitation. Protein samples from each fraction were stored at −20°C until further analysis.

### TFMS protein deglycosylation

Protein samples from the outer membrane fraction of different *H. pylori* strains were collected by subcellular fractionation and subjected to deglycosylation with a commercial deglycosylation kit (GlycoFree^TM^ Chemical Deglycosylation Kit, Prozyme, Santa Clara, CA, USA) according to the supplier’s protocol.

### Metabolic labeling and click chemistry-based methods

*H. pylori* 26695 wild-type strain was grown in Brucella broth as described previously with or without the addition of 1 mM tetraacetylated N-azidoacetylglucosamine (Ac_4_GlcNAz, Click-iT™ GlcNAz Metabolic Glycoprotein Labeling reagent, Invitrogen) for 4 d at 37°C, then harvested and lysed for total cell lysate preparation, or for subcellular fractionation. Total cell lysates or outer membrane samples from metabolically-labeled bacteria were subjected to the “click” reaction between an azide and an alkyne-biotin by employing the Click-iT™ Biotin Protein Analysis Detection Kit (Invitrogen) according to manufacturer’s instructions to detect the azide-labeled glycans. The resultant samples were used for immunoblotting analysis with peroxidase-labeled streptavidin (streptavidin-HRP, SeraCare KPL, Gaithersburg, MD, USA) to visualize biotin-tagged proteins, and with rabbit-anti-BabA polyclonal antibody to recognize BabA.

### N-linked deglycosylation with PNGase F

Deglycosylation with PNGase F was conducted according to the supplier’s protocol (New England Biolabs, Ipswich, MA, USA), with minor modifications. Briefly, 20 μg of protein sample from the outer membrane fraction of the wild-type strain 26695 were denatured at 100°C for 10 minutes. One microliter of PNGase F was added to the denatured protein sample and incubated at 37°C overnight for glycan digestion, and the obtained protein sample was analyzed using sodium dodecyl sulfate-polyacrylamide gel electrophoresis (SDS–PAGE) and immunoblotting.

### O-linked deglycosylation with β-elimination

Deglycosylation with β-elimination was conducted according to the supplier’s protocol (GlycoProfile^TM^ β-Elimination Kit, Sigma–Aldrich, St Louis, MO, USA). The obtained protein sample was analyzed by SDS–PAGE and immunoblotting.

### Fucosidase treatment

Enzymatic removal of fucose residues was conducted according to the supplier’s protocol (α1-3,4 Fucosidase, α1-2 Fucosidase, New England Biolabs) with minor modifications. Briefly, 20 μg of protein sample was used for the enzymatic treatment by adding 2 μl of the exoglycosidase. The resulting mixture was incubated at 37°C overnight for the removal of the fucose residues, followed by analyzing with SDS–PAGE and immunoblotting analysis.

### Protein electrophoresis and immunoblotting analysis

Protein samples were separated on acrylamide gels, with different concentrations ranging from 8% to 12%. SDS–PAGE was performed until the targeted adhesin migrated to the bottom edge of the gel for better resolution to clearly observe the differences in the molecular sizes of the tested adhesins. The separated proteins were transferred to 0.45 μm nitrocellulose membranes (Sartorius Stedim, Goettingen, Germany) for immunoblotting analysis. The obtained membranes were blocked with 5% skim milk in PBS or Tris-buffered saline (TBS) at room temperature for 1 h. After thorough washing, the blotted membranes were incubated at 4°C overnight with one of the following primary antibodies: rabbit-anti-AlpA polyclonal antibody, rabbit-anti-AlpB polyclonal antibody, rabbit-anti-BabA polyclonal antibody, rabbit-anti-BabB polyclonal antibody (adhesin-specific antibodies were kindly provided by Prof Rainer Haas from Max von Pettenkofer Institute), or mouse-anti-Lewis B antibody (Santa Cruz Biotechnology, Dallas, TX, USA).

### Two-dimensional sodium dodecyl sulfate-polyacrylamide gel electrophoresis (2D SDS-PAGE)

The protein samples prepared from the outer membrane fraction were desalted in a 2D lysis buffer (4% w/v 3-((3-cholamidopropyl) dimethylammonio)-1-propanesulfonate (CHAPS), 7 mol/L urea, 2 mol/L thiourea, 10 mmol/L Tris-HCl (pH 8.3), and 1 mmol/LEDTA) through a Nanosep membrane (Pall Corporation, New York City, NY, USA, molecular weight cutoff [MWCO] 10 kDa). After centrifugation at 10000 rpm for 15 min at 4°C, dithiothreitol (DTT) and immobilized pH gradient (IPG) buffer were added to the concentrated protein fractions. Subsequently, the nonlinear pH 3–10 IPG strips (GE Healthcare, Boston, MA, USA) were used to rehydrate the protein at room temperature for at least 12 h followed by isoelectronic focusing (IEF) of rehydrated protein through a Multiphor II Electrophoresis Unit for 62.5 kVh (GE Healthcare). After equilibrating the IPG strip with DTT and iodoacetamine (IAM), the equilibrated IPG strips were transferred onto 12.5% SDS-PAGE and separated by molecular weight. Subsequently, the separated proteins were electroblotted onto polyvinylidene difluoride (PVDF) membranes (Pall Corporation) followed by blocking with 5% w/v low fat milk in TBST for 1 h for later immunostaining analysis.

### LPS extraction and detection using silver staining and immunoblotting analysis

The wild-type strains 26695 and G27 and various mutant strains were collected, and the bacterial pellets were lysed. An equal amount of protein from each strain was treated with 15 μL of proteinase K (20 mg/mL) at 60°C for 2 h to degrade the protein. The obtained samples were then subjected to LPS extraction using the hot phenol-water method. An equal volume of 90% prewarmed phenol solution was added to the suspension and incubated in a 70°C water bath for 15 minutes with constant shaking per 5 minutes. The mixture was incubated on ice for 10 minutes before centrifugation (14,000*×*g, 10 minutes), and 100 μL of the supernatant (in the aqueous phase) were then collected for subsequent analyses. The obtained LPS samples from each strain was separated on 15% SDS–PAGE or Tricine-SDS–PAGE gels. For the immunoblotting analysis, the resulting gel was transferred to a 0.45 μm nitrocellulose membrane and probed with an anti-Lewis X antibody (Santa Cruz Biotechnology) or anti-Lewis Y antibody (Santa Cruz Biotechnology). For silver staining, the gel was fixed with 150 mL of the fixing buffer (25% isopropyl alcohol and 7.5% acetic acid) at 4°C overnight. The fixed gel was subjected to oxidation in 150 mL of the oxidation buffer (1.05 mg of periodic acid, 4 mL of alcohol, and 0.5 mL of acetic acid) at room temperature for 10 minutes, and then the gel was washed with 150 mL of distilled water three times for 30 minutes each. The gel was then stained with 150 mL of freshly prepared staining buffer (28 mL of 0.1 N NaOH, 2 mL of 29.4% ammonium hydroxide, and 5 mL of 20% silver nitrate) for 25 minutes. After thorough washing with 150 mL of distilled water three times for 10 minutes each, the gel was developed in 150 mL of the developing buffer (50 mg of citric acid and 0.5 mL of 37% formaldehyde).

### Protease sensitivity test

The protease sensitivity test was performed using two different procedures. *H. pylori* was cultured as described above. In the first procedure, bacteria were collected by centrifugation and then resuspended in 5 mL of PBS. Proteinase K was added to the suspensions to reach different final concentrations (0.5 μg/mL and 5 μg/mL). After an incubation on ice for 30 minutes, protease inhibitor (cOmplete^TM^ ULTRA Tablets, Roche, Basel, Switzerland) was added and incubated for 5 minutes to stop enzyme activity. The outer membrane samples were collected from proteinase K-treated bacteria by subcellular fractionation and then subjected to immunoblotting analysis. In the second procedure, the outer membrane samples were first extracted by subcellular fractionation. A fixed amount of outer membrane protein sample (10 µg) from each strain was treated with 0.5 μg/mL proteinase K in a time course analysis (0, 10, 30 minutes). After incubation on ice, a protease inhibitor (cOmplete^TM^ ULTRA Tablets) was added and incubated for 5 minutes to stop enzyme activity. The treated outer membrane samples were collected and then subjected to immunoblotting analysis.

### Protein stability assay

*H. pylori* was cultured as described above. Chloramphenicol was added to the *H. pylori* culture to a final concentration of 500 μg/mL and incubated for another 0, 3 or 6 h. A fixed volume of bacterial culture was then collected and subjected to subcellular fractionation. Outer membrane protein samples were collected for immunoblotting analysis.

### In-house ELISA of BabA-Lewis B antigen binding

Lacto-N-difucohexaose I (Lewis B)-BSA (Carbosynth, Berkshire, UK) was dissolved in PBS with 10% paraformaldehyde to a final concentration of 20 μg/mL. Fifty microliters of this solution were added to 96-well plates, and the plates were then exposed to ultraviolet light overnight at room temperature to fix the reactive complex on the plate wells. After fixation, the plates were washed twice with PBS. *H. pylori* was cultured as described above to an OD_600_ = 1.0. One microliter of bacterial culture was harvested by centrifugation, and the obtained pellet was resuspended in PBS with 2 μg/mL fluorescein isothiocyanate (FITC). After an incubation of 30 minutes at 37°C, bacteria were washed with PBS and then resuspended in TBS containing 2.5% BSA, 1 mM CaCl_2_, and 1 mM MgCl_2_ at different pH values (pH 7.0, pH 5.0, and pH 2.0). FITC-labeled bacteria derived from this reaction were added to the wells of Lewis B antigen-coated plates and incubated for 1 h with gentle agitation. For the antibody competition assay, FITC-labeled bacteria were coincubated with the BabA antibody at different dilution ratios (1:2000 and 1:1000) before being added to the wells of Lewis B antigen-coated plates. After incubation, the plates were first washed with TBST buffer twice and then incubated with a mouse anti-FITC monoclonal antibody (1:1000, Thermo Fisher Scientific, Waltham, MA, USA) for 1 h, followed by two washes with TBST buffer. Next, the HRP-conjugated goat anti-mouse antibody was added to the plates and incubated for 1 h, followed by two washes with TBST buffer. Then, 100 μL of trimethylborate substrate were added to each well of the plates, and the reaction was stopped by adding 50 μL of H_2_SO_4_. The binding between bacteria and Lewis B antigen was measured using an ELISA reader with an OD_450_ filter (Bio-Rad Laboratories, Hercules, CA, USA).

### Flow cytometry analysis of BabA-Lewis B antigen binding

One milliliter of bacterial culture (the wild-type strain 26695 and the *HP0859* knockout mutant) was harvested by centrifugation and adjusted to an OD_600_ = 0.1; the obtained suspension was then pelleted and washed twice with PBS. After thorough washing, bacteria were incubated with Lewis B antigen for 1 h in PBS containing 2.5% BSA, 1 mM CaCl_2_, and 1 mM MgCl_2_ to assess binding. After the incubation, bacteria bound to Lewis B antigen were incubated with an anti-Lewis B antibody (1:1000, Abcam, Cambridge, UK) for 1 h, followed by an incubation with the corresponding secondary antibody conjugated to FITC (1:1000) for 30 minutes for recognition. Between and after incubation, bacteria were washed with PBS twice. The resulting samples were analyzed using flow cytometry according to the user manual of BD Accuri^TM^ C6 PLUS (BD, East Rutherford, NJ, USA). The fluorescence of Lewis B antigen-bound bacteria was calibrated based on the signal of bacteria without Lewis B antigen binding as the control.

### Adhesion assays

*H. pylori* was cultured as described above for 24 h. Bacteria were collected by centrifugation and resuspended in Ham’s F12 medium supplemented with 10% FBS to an OD_600_ = 0.5. The bacterial suspension was then used for the infection process. The AGS cells were cultured in Ham’s F12 medium supplemented with 10% FBS and then seeded in 12-well plates. The bacterial suspension was then added to the wells of the AGS cells at an MOI of 100 and incubated for 6 h under standard culture conditions. The nonadherent bacteria were removed by washing with PBS, and then the cells were lysed with 0.5% saponin-containing PBS for 5 minutes. The resulting lysate was serially diluted and spread on sheep's blood agar plates. The adherent bacteria were measured using a viable plate counting method after 72–96 h of incubation. The experiment was repeated in triplicate to obtain the average and the standard deviation.

### Molecular cloning of the *babA* gene and its expression in *E.* *coli*

The *babA* gene was amplified from the wild-type strain 26695 genomic DNA as a template (Supplementary Table S3) using *Pfu* DNA polymerase (Promega, Madison, WI, USA). The PCR product was ligated into a pGEM-T vector (Promega), and the resulting plasmid was transformed into *E. coli* Top10 competent cells. The plasmid containing the correct *babA* gene sequence was digested with restriction enzymes *Nco*I and *Xho*I and the obtained fragment was then subcloned into the expression vector pET28a (Novagen, Darmstadt, Germany), followed by transformation into *E. coli* BL21 (DE3) competent cells. The transformed cells were selected from agar plates containing the antibiotic kanamycin and subjected to subcellular fractionation, SDS–PAGE, and immunoblotting analysis.

### Statistical analysis

Statistical analyses were performed using the unpaired, two-tailed Student’s *t*-test. Significant differences were identified at *p < .05, **p < .01 and ***p < .001.

## Supplementary Material

Supplemental Material

## Data Availability

Data sharing is not applicable to this article as no new data were created or analyzed in this study.
