# Supplementary material for: Helicobacter pylori employs a general protein glycosylation system for the modification of outer membrane adhesins
Source: Gut Microbes. 2022 Oct 7;14(1):2130650. doi: 10.1080/19490976.2022.2130650 (PMC9553153; doi:10.1080/19490976.2022.2130650)
Supplement: Supplemental Material [file KGMI_A_2130650_SM2783.zip › KGMI-20220011R2-Supplementary file.docx]

**SUPPLEMENTARY MATERIAL**

*Helicobacter pylori* employs a general protein glycosylation system for the modification of outer membrane adhesins

Kai-Wen Teng^1^, Kai-Siang Hsieh^1^, Ji-Shiuan Hung^1^, Chun-Jen Wang^1^, En-Chi Liao^2,3^, Pei-Chun Chen^1^, Ying-Hsuan Lin^1^, Deng-Chyang Wu^4^, Chun-Hung Lin^5^, Wen-Ching Wang^6,7^, Hong-Lin Chan^2,3^, Shau-Ku Huang^8^, Mou-Chieh Kao^1,7*^

^*^Corresponding author: Mou-Chieh Kao Ph.D.

Email: mckao@life.nthu.edu.tw

**This PDF file includes:**

Figures S1 to S5

Tables S1 to S3

**SUPPLEMENTARY FIGURES**


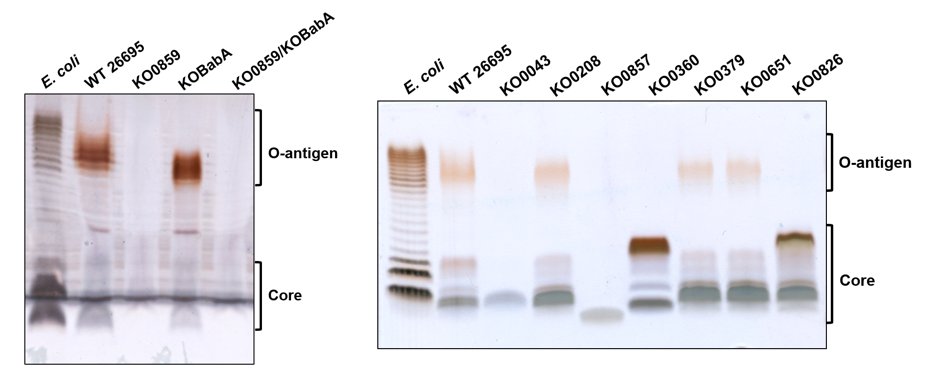


**Supplementary Figure S1.** The LPS profiles of various *H. pylori* LPS-related knockout mutants were evaluated using silver staining. LPS samples were isolated from the total cell lysates of various *H. pylori* strains. LPS samples from the wild-type strain 26695 and the *E. coli* O111:B4 were applied as the controls for comparison. WT, wild-type strain; KO, knockout mutant.


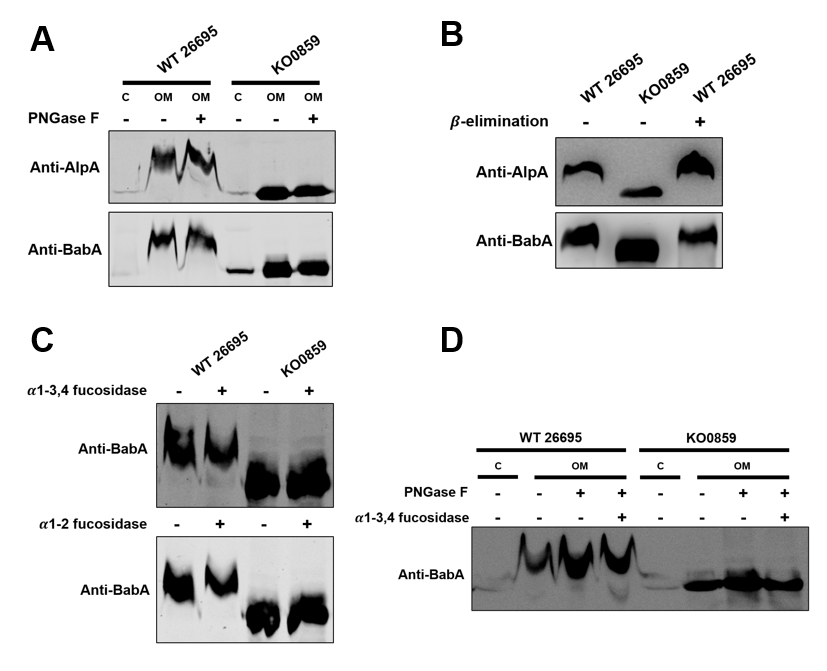


**Supplementary Figure S2.** The molecular sizes of key adhesins remained unchanged after enzymatic or chemical deglycosylation processes. Electrophoretic migration of the tested adhesins. (**A**) Outer membrane protein samples from both the wild-type strain 26695 and the *HP0859* knockout mutant were treated with or without PNGase F for N-linked enzymatic deglycosylation, and the electrophoretic migration patterns of adhesins AlpA and BabA were compared using immunoblotting with AlpA- and BabA-specific antibodies. (**B**) Outer membrane protein samples from the wild-type strain 26695 were subjected to β-elimination treatment, and the electrophoretic migration patterns of adhesins AlpA and BabA were compared using immunoblotting with AlpA- and BabA-specific antibodies. Outer membrane protein samples from the wild-type strain 26695 and the *HP0859* knockout mutant without β-elimination treatment were applied as the controls. (**C**) Outer membrane protein samples from both the wild-type strain 26695 and the *HP0859* knockout mutant were treated with or without $\alpha$1-3,4 fucosidase and $\alpha$1-2 fucosidase for the removal of fucose residues, and the electrophoretic migration pattern of BabA was compared using immunoblotting with BabA-specific antibodies. (**D**) Outer membrane protein samples from both the wild-type strain 26695 and the *HP0859* knockout mutant were treated with or without PNGase F and $\alpha$1-3,4 fucosidase for glycan removal, and the electrophoretic migration pattern of BabA was compared using immunoblotting with BabA-specific antibodies. Cytosolic and outer membrane protein samples from the wild-type strain 26695 and the *HP0859* knockout mutant without the enzymatic treatment were applied as the controls. Protein samples from each subcellular fraction were analyzed using 12$\%$ SDS–PAGE gels for AlpA separation or by 10$\%$ SDS–PAGE gels for BabA separation. WT, wild-type strain; KO, knockout mutant; C, cytoplasm; OM, outer membrane.


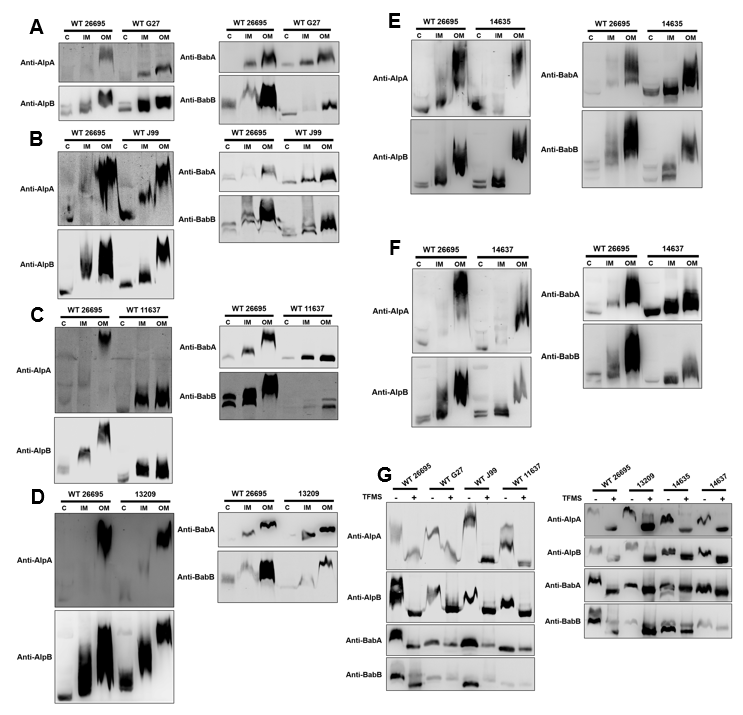


**Supplementary Figure S3.** Key adhesins from other sequenced *H. pylori* strains and clinically isolated strains are modified by glycans. Electrophoretic migration of the tested adhesins. (**A** to **F**) The electrophoretic migration patterns of key adhesins from (**A**) sequenced strain G27, (**B**) sequenced strain J99, (**C**) sequenced strain NCTC 11637, (**D**) clinical strain 13209, (**E**) clinical strain 14635 and (**F**) clinical strain 14637 were evaluated using immunoblotting with AlpA-, AlpB-, BabA- and BabB-specific antibodies. The wild-type strain 26695 served as the control. Protein samples were isolated from the cytoplasm (C), inner membrane (IM) and outer membrane (OM) of bacteria. (**G**) Outer membrane protein samples from the tested *H. pylori* strains were deglycosylated by TFMS and then subjected to immunoblotting with AlpA-, AlpB-, BabA- and BabB-specific antibodies. Protein samples from each subcellular fraction were analyzed using 12$\%$ SDS–PAGE gels for AlpA/B separation or 10$\%$ SDS–PAGE gels for BabA/B separation. WT, wild-type strain.


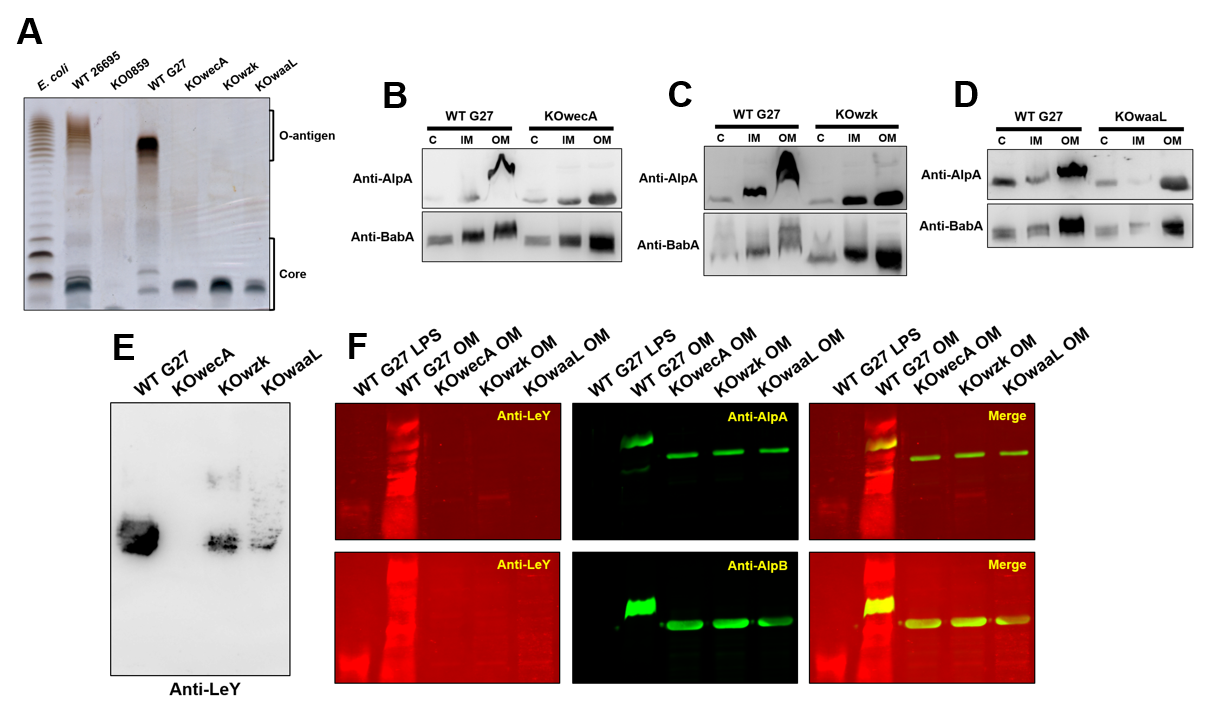


**Supplementary Figure S4.** Disruption of the LPS O-antigen biosynthesis pathway in the wild-type strain G27 alters the electrophoretic migration patterns of key adhesins and the decoration of Lewis Y antigen on AlpA/B. (**A**) The successful generation of LPS O-antigen biosynthesis-disrupted mutants was confirmed by performing silver staining to reveal the LPS profiles of mutants. LPS samples were isolated from the total bacterial cell lysates. LPS samples from the wild-type strain 26695 and the *HP0859* knockout mutant were applied as the controls for comparison. The electrophoretic migration patterns of the tested adhesins from (**B**) *wecA*, (**C**) *wzk* and (**D**) *waaL* knockout mutants were compared to those of the wild-type strain G27 using immunoblotting with AlpA- and BabA-specific antibodies. (**E**) Decoration of Lewis Y antigen (LeY) on the O-antigen terminus of the wild-type strain G27 was detected using immunoblotting with an anti-Lewis Y antibody. LPS samples were isolated from the total bacterial cell lysates and subjected to analysis. (**F**) Evaluation of signal overlap for AlpA/B with Lewis Y antigen. The overlapping signals for AlpA/B and Lewis Y antigen were detected using immunoblotting and visualized using the fluorescent gel scan method (red: 700 nm emission, Lewis Y antigen; green: 800 nm emission, AlpA/B). Protein samples isolated from the cytoplasm (C), inner membrane (IM) and outer membrane (OM) of bacteria were analyzed using 12% SDS–PAGE gels for AlpA separation or 10% SDS–PAGE gels for BabA separation. LPS samples from the wild-type strain G27 were used as a control. WT, wild-type strain; KO, knockout mutant.


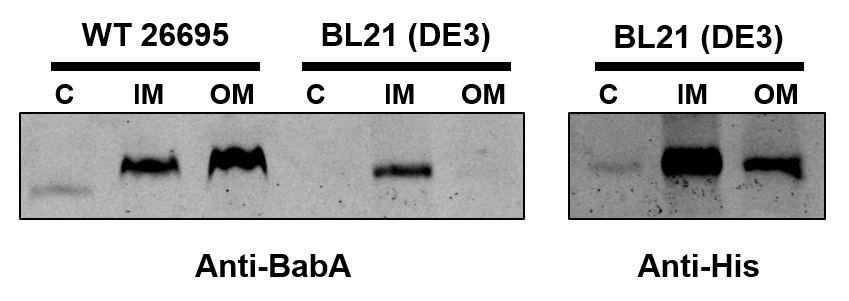


**Supplementary Figure S5.** The exogenously-expressed BabA protein is not modified by glycans. Protein samples derived from subcellular fractionation of the *H. pylori* wild-type strain 26695 and the *E. coli* strain BL21 (DE3) carrying a plasmid for heterologous expression of BabA were analyzed using immunoblotting with anti-BabA and anti-His antibodies, and the electrophoretic migration patterns of BabA were compared. Protein samples from each subcellular fraction were analyzed using 10$\boldsymbol{\%}$ SDS–PAGE gels for BabA separation. WT, wild-type strain; C, cytoplasm; IM, inner membrane; OM, outer membrane.

**SUPPLEMENTARY TABLES**

**Supplementary Table S1.** List of LPS biosynthesis-related enzymes disrupted in this study and their corresponding function.

| **No.** | **26695 ORF^a^/G27 ORF** | **Encoded enzyme** | **Enzyme function** | |
| --- | --- | --- | --- | --- |
| **LPS biosynthesis** | | | | |
| **1** | HP0093/94 | FutC, $\alpha$-1,2-fucosyltransferase | Lewis antigen construction |  |
| **2** | HP0379 | FutA, $\alpha$-1,3-fucosyltransferase | Lewis antigen construction |  |
| **3** | HP0651 | FutB, $\alpha$-1,3-fucosyltransferase | Lewis antigen construction |  |
| **4** | HP0826 | $\beta$-1,4-Gal transferase | Lewis antigen construction |  |
| **5** | HP1039/HPG27_389 | WaaL, O-antigen ligase | O-antigen transfer |  |
| **6** | HP1206/HPG27_1153 | Wzk, O-antigen flippase | O-antigen flip |  |
| **7** | HP1581/HPG27_1518 | WecA, UDP-GlcNAc transferase | GlcNAc transfer |  |
| **Monosaccharide biosynthesis (also included in LPS biosynthesis)** | | | | |
| **8** | HP0043 | GDP-mannose pyrophosphorylase | Fucose biosynthesis | |
| **9** | HP0360 | UDP-glucose-4-epimerase | Galactose biosynthesis | |
| **10** | HP0858 | bifunctional heptose 7-phosphate kinase/heptose 1-phosphate adenylyltransferase | Heptose biosynthesis | |
| **11** | HP0859 | ADP-D-glycero-D-manno-heptose-6-epimerase | Heptose biosynthesis | |
| **Putative enzyme** | | | | |
| **12** | HP0208 | Glycosyltransferase | -- | |

^a^ORF, open reading frame.

**Supplementary Table S2.** Sequenced *H. pylori* strains and clinically isolated strains used in this study.

| **Strain** | **Genotype** | **Origin** | **Clinical diagnosis** | **Source** |
| --- | --- | --- | --- | --- |
| **Sequenced strain** | | | | |
| 26695 | CagA^+^, VacA^+^ | United Kingdom | Gastritis | Tomb et al. |
| G27 | CagA^+^, VacA^+^ | Italy | Unknown disease | Baltrus et al. |
| J99 | CagA^+^, VacA^+^ | USA | Duodenal ulcer | Alm et al. |
| NCTC 11637 | CagA^+^, VacA^+^ | Australia | Unknown disease | Clancy et al. |
| **Clinically isolated strain** | | | | |
| 13209 | ND^a^ | Taiwan | Duodenal ulcer | This study |
| 14635 | ND | Taiwan | Gastric cancer phase IIIc | This study |
| 14637 | ND | Taiwan | Gastric cancer phase III | This study |

^a^ND, not determined.

**Supplementary Table S3.** List of primers used in this study.

| **Name** | **Sequence^a^** | **Restriction site** |
| --- | --- | --- |
| **KO0043 F** | 5’-atgcgacatatgaaaattaaaaatatctt-3’ | *Nde*I |
| **KO0043 R** | 5’-gagcctcgagagccagatagctcccgtctt-3’ | *Xho*I |
| **KO0043 mF** | 5’-ctcgttggatccaaaagagc-3’ | *Bam*HI |
| **KO0043 mR** | 5’-gctcttttggatccaacgag-3’ | *Bam*HI |
| **KO0093/94 F** | 5’-catatggcttttaaggtggtgc-3’ | *Nde*I |
| **KO0093/94 R** | 5’-gacgctcgagataaagaaatcc-3’ | *Xho*I |
| **KO0093/94 mF** | 5’-aaaccgaggatccggcaaagcgcgt-3’ | *Bam*HI |
| **KO0093/94 mR** | 5’-ccggatcctcggtttgcttgattaaag-3’ | *Bam*HI |
| **KO0208 F** | 5’-atgcgacatatgcaagagattatccctat-3’ | *Nde*I |
| **KO0208 R** | 5’-gagcctcgagaaggagatccagcccac-3’ | *Xho*I |
| **KO0208 mF** | 5’-tatacggatcccgccaaatg-3’ | *Bam*HI |
| **KO0208 mR** | 5’-catttggcgggatccgtata-3’ | *Bam*HI |
| **KO0360 F** | 5’-atgcgacatatggcattattattcacagg-3’ | *Nde*I |
| **KO0360 R** | 5’-gagcctcgaggctcgctgaaattcaagcac-3’ | *Xho*I |
| **KO0360 mF** | 5’-aagcccctggatccccatta-3’ | *Bam*HI |
| **KO0360 mR** | 5’-taatggggatccaggggctt-3’ | *Bam*HI |
| **KO0379 F** | 5’-atgcgacatatgttccaacccctattag-3’ | *Nde*I |
| **KO0379 R** | 5’-gagcctcgagtagcgcaaggggtttgatta-3’ | *Xho*I |
| **KO0379 mF** | 5’-cagtttggatccgagcaacg-3’ | *Bam*HI |
| **KO0379 mR** | 5’-cgttgctcggatccaaactg-3’ | *Bam*HI |
| **KO0651 F** | 5’-atgcgacatatgttccaacccctattagacg-3’ | *Nde*I |
| **KO0651 R** | 5’-gagcctcgagcaaaaaccccacgctcaa-3’ | *Xho*I |
| **KO0826 F** | 5’-gtaccatatgttgcgtgtttttgccatt-3’ | *Nde*I |
| **KO0826 R** | 5’-gagcctcgagaaggcggttaagttttgttc-3’ | *Xho*I |
| **KO0826 mF** | 5’-tgggtggatcccgagtcat-3’ | *Bam*HI |
| **KO0826 mR** | 5’-atgactcgggatccaccca-3’ | *Bam*HI |
| **KO0858 F1** | 5’-tcttagtcataggcgatctg-3’ | - |
| **KO0858 R1** | 5’-gagttcaaaggatccaacac-3’ | *Bam*HI |
| **KO0858 F2** | 5’-gtgttggatcctttgaactc-3’ | *Bam*HI |
| **KO0858 R2** | 5’-tcttctaaactcgctaacgc-3’ | - |
| **P_HP1563_ F1** | 5’-agtaccatggttgacttggatttc-3’ | *Nco*I |
| **P_HP1563_-HP0858 Com R1** | 5’-cgcctatgactaagatttttttcatatcgtaactcc-3’ | - |
| **HP0858 F** | 5’-ggagttacgatatgaaaaaaatcttagtcataggcg-3’ | - |
| **HP0858 R** | 5’-agtaggtacctcaatcattgc-3’ | *Kpn*I |
| **KO0859 F** | 5’-atgcgacatatgcgttatattgatgatgaatta-3’ | *Nde*I |
| **KO0859 R** | 5’-gagcctcgagaaagccgttcgctcttcc-3’ | *Xho*I |
| **KO0859 mF** | 5’-tggtaggatccaacgaaagc-3’ | *Bam*HI |
| **KO0859 mR** | 5’-gctttcgttggatcctacca-3’ | *Bam*HI |
| **P_ureA_ F1** | 5’-actgccatggagtcgtggccaccattatc-3’ | *Nco*I |
| **P_ureA_-HP0859 Com R1** | 5’-ggggccgcggcttattctcctattcttaaa-3’ | *Sac*II |
| **HP0859 F** | 5’-cgatccgcggatgcgttatattgatgatg-3’ | *Sac*II |
| **HP0859 R** | 5’-ctgctcgagaaagccgttcgctcttcc-3’ | *Xho*I |
| **KO1039 F1** | 5’-tcattccagtcgtttagggc-3’ | - |
| **KO1039 R1** | 5’-gaaaggatccgtttggctatcatg-3’ | *Bam*HI |
| **KO1039 F2** | 5’-aaacggatcctttccgctacaatt-3’ | *Bam*HI |
| **KO1039 R2** | 5’-tccccacatacaaagcgctc-3’ | - |
| **KO1206 F1** | 5’-cctgcttgtgctgatggctg-3’ | - |
| **KO1206 R1** | 5’-ttttggatcccttgatgagaacggtg-3’ | *Bam*HI |
| **KO1206 F2** | 5’-caagggatccaaaaagggtgagatgg-3’ | *Bam*HI |
| **KO1206 R2** | 5’-ttctgacctctttgaatggtgagg-3’ | - |
| **KO1581 F1** | 5’-catcatgcaagaaccccacg-3’ | - |
| **KO1581 R1** | 5’-caagggatcccaccatgtaagctaat-3’ | *Bam*HI |
| **KO1581 F2** | 5’-ggtgggatcccttgggtttatggtgt-3’ | *Bam*HI |
| **KO1581 R2** | 5’-gcacaaacttgcctgttcaaataagc-3’ | - |
| **KOBabA F1** | 5’-acaccaaaggcatccaacag-3’ | - |
| **KOBabA R1** | 5’-tataggatccggttacactgacctt-3’ | *Bam*HI |
| **KOBabA F2** | 5’-aaccggatcctatacctacacatgc-3’ | *Bam*HI |
| **KOBabA R2** | 5’-atggcttgccccacctgatg-3’ | - |
| **EC-BabA-His-F** | 5’-aaaacccatgggcaaaaaacacatc-3’ | *Nco*I |
| **EC-BabA-His-R** | 5’-aacactcgaggtaagcgaacacat-3’ | *Xho*I |

^a^ The sequences of restriction enzyme sites are underlined.
