# Supplementary figures and images for: Helicobacter pylori employs a general protein glycosylation system for the modification of outer membrane adhesins
Source: Gut Microbes. 2022 Oct 7;14(1):2130650. doi: 10.1080/19490976.2022.2130650 (PMC9553153; doi:10.1080/19490976.2022.2130650)

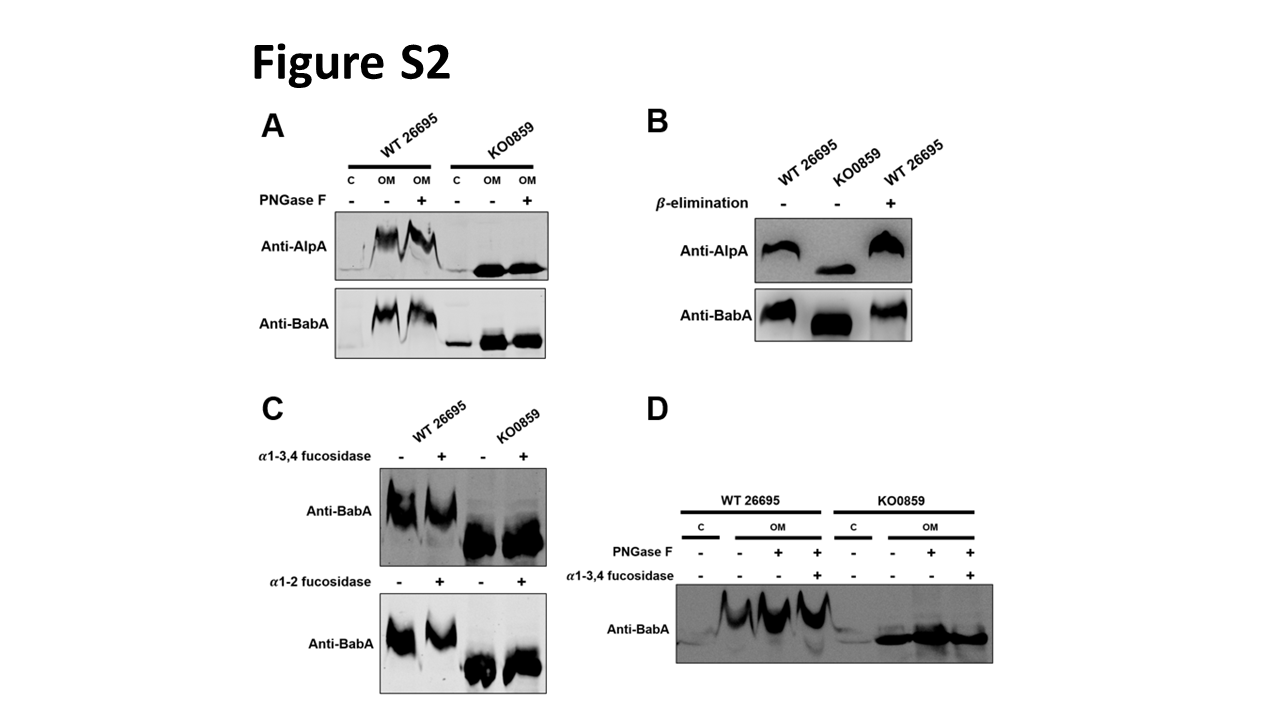

Supplement: Supplemental Material [file KGMI_A_2130650_SM2783.zip › KGMI-20220011R2-Fig S2.tif]

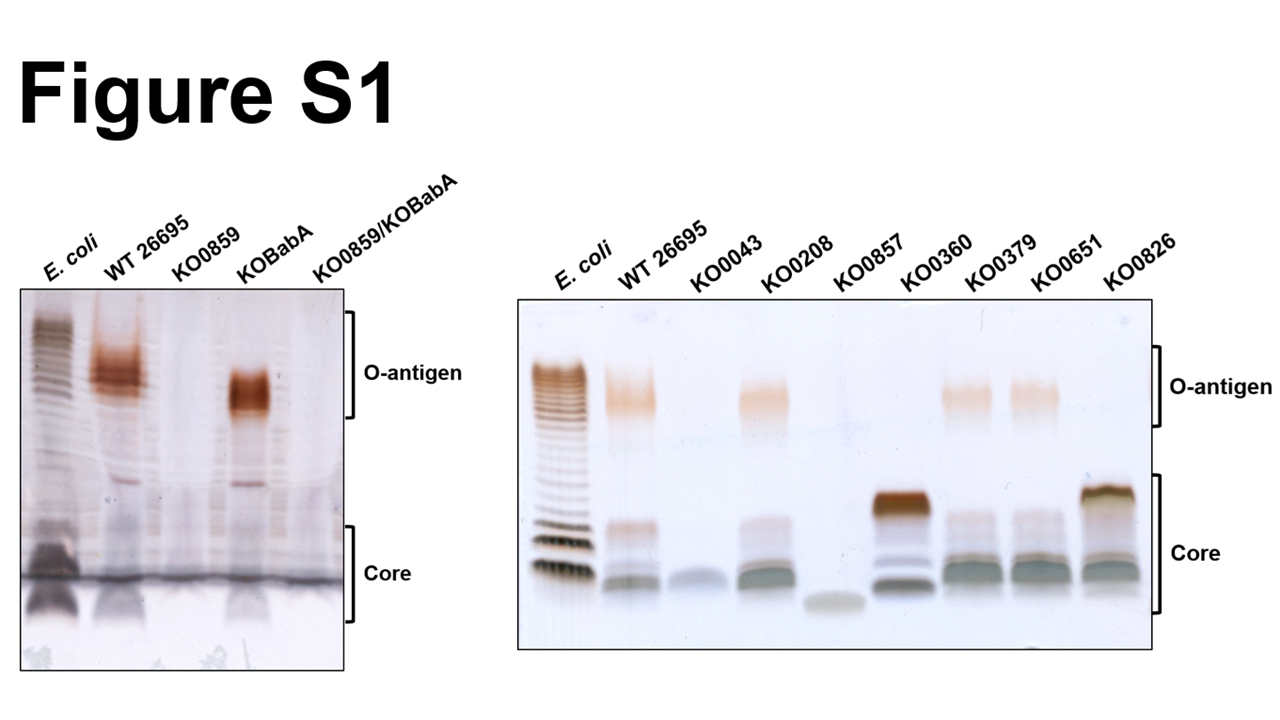

Supplement: Supplemental Material [file KGMI_A_2130650_SM2783.zip › KGMI-20220011R2-Figure S1.tif]

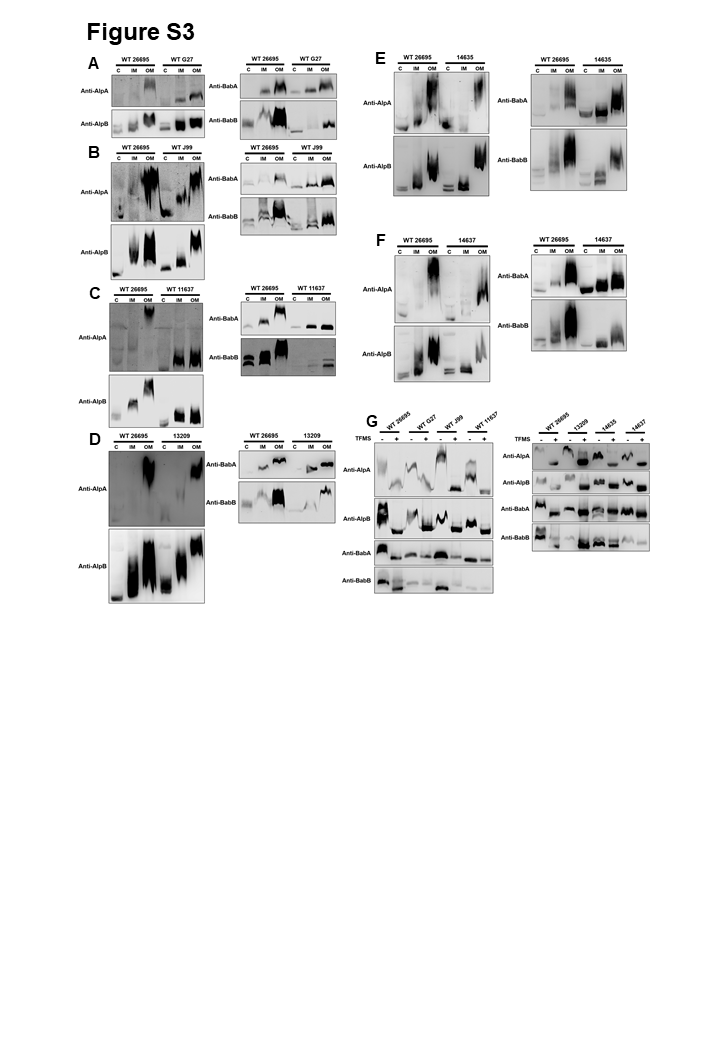

Supplement: Supplemental Material [file KGMI_A_2130650_SM2783.zip › KGMI-20220011R2-Figure S3.tif]

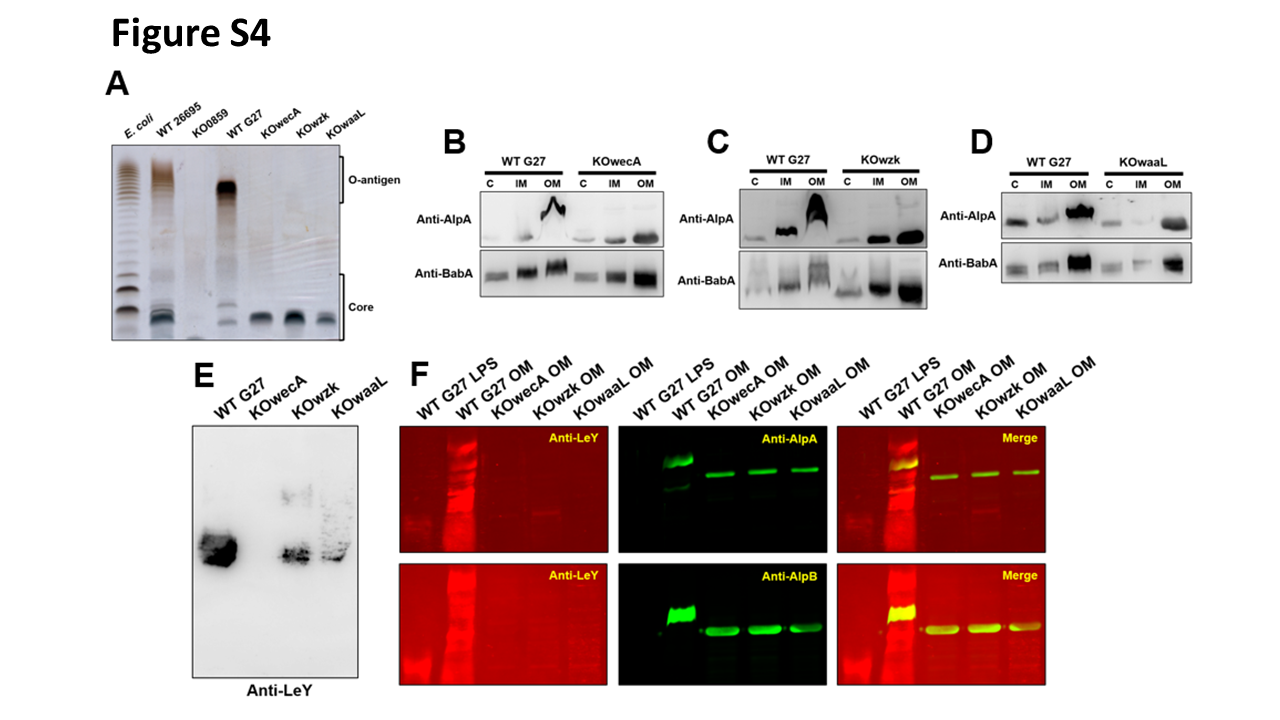

Supplement: Supplemental Material [file KGMI_A_2130650_SM2783.zip › KGMI-20220011R2-Figure S4.tif]

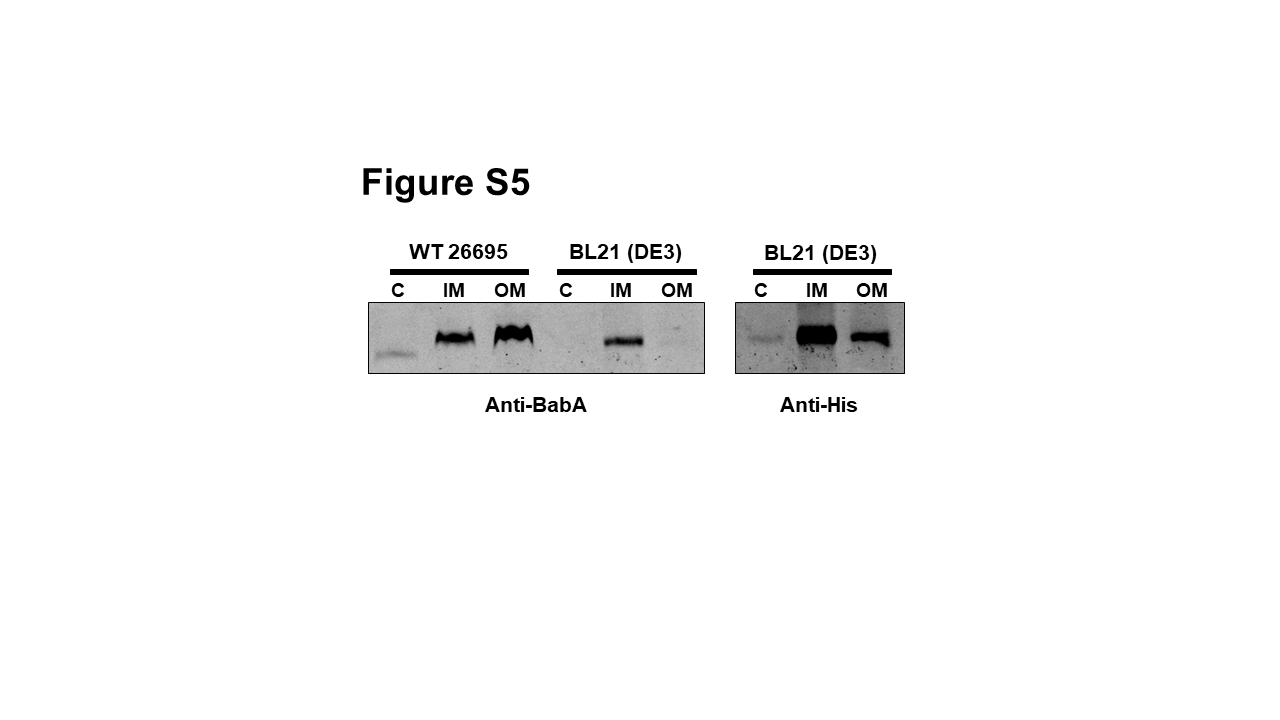

Supplement: Supplemental Material [file KGMI_A_2130650_SM2783.zip › KGMI-20220011R2-Figure S5.tif]
